# Molecular Informatics, Chemometrics, and Sensory Omics for Constructing an Umami Peptide Cluster Library Across the Entire Lager Beer Brewing Process

**DOI:** 10.3390/foods15040641

**Published:** 2026-02-10

**Authors:** Yashuai Wu, Ruiyang Yin, Wenjing Tian, Wanqiu Zhao, Jiayang Luo, Mingtao Huang, Dongrui Zhao

**Affiliations:** 1 School of Food Science and Engineering, South China University of Technology, Guangzhou 510640, China; wyss995418706@163.com (Y.W.); huangmt@scut.edu.cn (M.H.); 2 Technology Center of Beijing Yanjing Beer Co., Ltd., Beijing 101300, China; ruiyang_yin@163.com; 3 Department of Food and Bioengineering, Beijing Vocational College of Agriculture, Beijing 102442, China; 91505@bvca.edu.cn (W.T.); 18401344071@163.com (W.Z.); b230690090@163.com (J.L.); 4 China Food Flavor and Nutrition Health Innovation Center, Beijing Technology and Business University, Beijing 100048, China; 5 Key Laboratory of Brewing Molecular Engineering of China Light Industry, Beijing Technology & Business University, Beijing 100048, China; 6 Beijing Laboratory of Food Quality and Safety, Beijing Technology and Business University, Beijing 100048, China

**Keywords:** lager beer, umami peptide cluster, T1R1/T1R3, molecular informatics, chemometrics, sensory omics

## Abstract

Umami taste in lager beer not only determined body fullness and the backbone of aftertaste, but also affected the controllability and interpretability of flavor expression across the entire brewing process. Based on stage-wise sampling, peptidomic profiles were established on wort fermentation day 0, day 1, day 3, and day 9. A total of 25,592 peptides were identified by reversed-phase liquid chromatography–quadrupole time-of-flight mass spectrometry (RPLC-QTOF-MS). Molecular informatics screening was performed using UMPred-FRL (a feature representation learning-based meta-predictor for umami peptides) and TastePeptides-Meta (a one-stop platform for taste peptides and prediction models), yielding 7255 potential umami peptides. From these, 145 peptides were further selected for molecular docking. In addition, 6 representative umami peptides were selected for receptor-level validation and structural analysis. Mechanistically, the umami receptor taste receptor type 1 member 1/taste receptor type 1 member 3 (T1R1/T1R3) belonged to class C G protein-coupled receptor (GPCR) and relied on the extracellular Venus flytrap (VFT) domain for ligand capture. Ligand-induced VFT conformational convergence transmitted changes to the transmembrane region and triggered signal transduction. Docking and energy decomposition indicated that the ionic group primarily contributed to orientation and anchoring. Salt-bridge or hydrogen-bond networks were formed around Lys228, Arg240, Glu206, Asp210, Asn141, and Gln138, thereby reducing conformational freedom. Meanwhile, hydrophobic side chains obtained major binding gains within a hydrophobic microenvironment formed by Val135, Ile137, Leu165, Tyr166, Trp78, and His79. These results reflected a synergistic mode in which charge pairing enabled positioning and hydro-phobic complementarity promoted VFT closure. To experimentally confirm sensory relevance, 6 representative peptides were individually spiked into 4 brewing-stage beer samples, which produced a clear stratification pattern across stages. Notably, peptides with favorable docking-derived binding propensity did not necessarily enhance umami perception, and several longer peptides showed persistent negative sensory shifts, supporting that binding affinity alone could not be treated as a proxy for perceived umami in the beer matrix. At the node level, the cumulative abundance of umami peptides showed a significant positive correlation with umami scores, with a Pearson correlation coefficient of r = 0.963 and *p* = 0.037. This result indicated good linear consistency between umami peptide content and the upward shift in umami taste in lager beer. Umami peptide clusters were further proposed as a more appropriate functional unit, and an umami peptide cluster database spanning the full process was constructed. This database provided a reusable resource for process control and flavor prediction.

## 1. Introduction

Lager beer was one of the most consumed alcoholic beverages worldwide. It was favored for its crisp mouthfeel and the balanced malt and hop aroma. This light and refreshing profile also made lager beer a common choice for cooling and thirst relief. Lager beer also contained nutrients and functional components derived from malt and hops, including B vitamins, silicon, and polyphenolic antioxidants [1,2]. Under moderate consumption, these components were considered potentially beneficial to human health. The combination of distinctive sensory experience and potential nutritional value supported a broad consumer base worldwide [3,4].

The large market scale of lager beer further reflected its popularity. In 2024, the global beer market was valued at approximately USD 804.65 billion. Lager beer, including pale, Vienna, and dark styles, accounted for about 86.46% of the market, with sales of approximately USD 695.7 billion [5]. Overall, lager beer represented close to 90% of global beer volume. This pattern indicated that lager beer remained the dominant beer type across regions. High consumption volume and market share also suggested strong public acceptance and stable flavor quality [6,7,8].

During long-term beer flavor research, umami was gradually recognized as a component of lager beer flavor quality. Umami provided a mellow and full-bodied taste and was considered an important factor in improving overall flavor quality. However, compared with sweetness, sourness, and bitterness, mechanistic research on umami characteristics in beer remained at an early stage. The molecular basis of beer umami sources also remained insufficiently clear. In other words, the presence and importance of umami in beer had been noted, but its material basis and mechanisms still required systematic elucidation [4,5,9,10].

In foods, umami perception was typically provided by small-molecule compounds. Free amino acids, including L-glutamate and L-aspartate and their sodium salts, were widely present in nature. 5′-nucleotides, including inosinate (IMP) and guanylate (GMP), also exhibited umami characteristics. Organic acids and carboxylic acids were also associated with umami. Oligopeptides were another important contributor [10,11,12,13]. These low-molecular-weight peptides could also exhibit umami or synergistically enhance umami. During food processing, including fermentation, enzymatic hydrolysis, and thermal processing, precursors such as proteins and nucleic acids were transformed into these umami-related molecules. This transformation provided a savory basis for soups, sauces, and fermented alcoholic beverages. Similarly, during mashing, yeast fermentation, and maturation in brewing, these umami-active substances were gradually accumulated and contributed to an umami background in the final product [14,15,16,17].

Among various umami substances, umami peptides have attracted increasing attention due to their unique roles. Umami peptides were generally composed of several amino acids linked by peptide bonds, and their molecular weight was usually below 3 kDa. Because some sequences were rich in glutamate or aspartate residues, these peptides could bind to human umami receptors and produced notable umami-enhancing effects. Studies indicated that umami-active peptides were widely present in animal-derived foods, plant-derived foods, and fermented foods [18,19,20,21]. In many traditional fermented products, including cheese, soy sauce, fermented ham, rice wine, beer, and Japanese sake, a series of peptides with umami or rich flavor characteristics had been isolated and identified. These findings suggested that small peptides acted as emerging flavor substances and played important roles in enhancing umami and improving quality [22,23,24].

Many food studies had identified representative umami peptides and confirmed their contribution to flavor. For example, an octapeptide first discovered in beef broth, with the sequence Lys-Gly-Asp-Glu-Glu-Ser-Leu-Ala, was verified to exhibit a strong umami taste [25,26]. In soy sauce, a series of dipeptides with pyroglutamate at the N-terminus, including pGlu-Glu, pGlu-Pro, and pGlu-Ile, were reported, with a total content up to 770 mg/L [27,28,29]. These dipeptides contributed substantially to the intense umami of soy sauce. In addition, an octapeptide isolated from pufferfish muscle, Tyr-Gly-Gly-Thr-Pro-Pro-Phe-Val, was reported [30]. This peptide not only imparted umami, but also showed a slight sweetness. These examples indicated that small peptides from different food sources could markedly enhance savory flavor and were closely linked to raw materials and processing.

In contrast, newly formed umami peptides during beer brewing were more complex, and related research remained an emerging field. Brewing involved multiple stages, including malt mashing, bottom fermentation, and storage maturation. During these stages, raw-material components underwent substantial changes under the action of yeast and enzymes, and a complex flavor matrix was formed. Importantly, beer-specific literature has shown that proteins, polypeptides, and their derived peptide fractions are not only analytical targets but also key determinants of overall beer quality, because they contribute to colloidal structure and thereby shape body, smoothness, and mouthfeel perception. Experimental fractionation studies further supported this sensory relevance by demonstrating that distinct molecular-weight peptide and polypeptide pools modulated different mouthfeel dimensions, including improved smoothness and softness, reduced astringency, and enhanced palate fullness and body, with low-molecular-weight polypeptides being associated with increased body and umami-like taste attributes in beer [31]. This multistage and multifactor process led to diverse and complex mechanisms for the generation of taste-active peptides. A limited number of studies indicated that protein hydrolysis during malt preparation and fermentation could release many small peptides [32,33]. Short peptides rich in glutamate or aspartate residues were suggested to directly contribute to umami in beer. For example, LC-MS/MS analysis detected more than 200 low-molecular-weight peptides in commercial barley malt beer. Several peptides contained acidic amino-acid motifs and were inferred to activate umami receptors. Other reports indicated that after aging on yeast autolysate material (lees), free glutamate and small-peptide contents increased markedly. Matured beer samples therefore exhibited higher umami potential than fresh beer. Taken together, these beer-specific findings suggested that umami peptides should be discussed within a broader sensory framework, because peptide formation may simultaneously influence receptor-mediated umami signaling and the matrix-driven attributes of body and smoothness that govern drinking quality. Although these findings suggested a relationship between peptide formation and umami gain during fermentation and maturation, identification and functional studies of umami peptides in beer remained limited [34,35]. Specific peptides responsible for major umami effects in beer had not been clearly established. Therefore, profiling and mining umami peptide composition across the full brewing process remained important for elucidating the formation mechanism of beer umami and for better linking umami-active candidates to beer-relevant sensory quality dimensions such as body, mouthfeel, and smoothness.

To address this gap, this study was designed to systematically investigate the composition and dynamic changes in umami peptides throughout lager beer brewing. Samples were collected at 4 key time points, wort fermentation day 0, day 1, day 3, and day 9. These time points represented unfermented wort, early fermentation, mid fermentation, and late fermentation near the completion of primary fermentation. Samples spanning the start and end of fermentation were used to capture the dynamic transition of umami peptides from absence to emergence and from low to higher abundance. Potential umami peptides arising during the brewing process were then identified. To comprehensively mine umami peptides in lager beer, chemometrics and sensomics were integrated. First, RPLC-QTOF-MS was applied to analyze peptide profiles across fermentation stages and to screen peptide molecules present in lager beer. Subsequently, an umami peptide prediction model was used to simulate the binding affinity between candidate peptides and the umami receptor (T1R1/T1R3). Umami potential was also estimated. Specific peptide segments with potential umami activity were screened. Molecular docking was applied to validate the molecular mechanism. Finally, a modern sensomics approach was used to evaluate the sensory impact of umami peptides on beer body. An umami peptide cluster database covering the full lager beer brewing process was established. Using this integrated strategy, umami peptides generated at each brewing stage were systematically profiled and their roles were clarified. These outcomes provided scientific evidence for the molecular mechanism underlying umami formation in beer. They also offered new approaches for improving beer quality through flavor optimization.

## 2. Materials and Methods

### 2.1. Samples and Reagents

The experimental samples were craft lager beer with an original wort extract of 8 °P. To better specify the beer formulation for peptide interpretation, the grist was defined on a weight basis as malted barley as the primary source (approximately 70–90% of total grist), with rice as the main adjunct (approximately 10–20%), and a minor wheat fraction included to modulate foam and mouthfeel (typically 0–10% within the total grist, depending on batch design). In the present formulation, malted barley, wheat, and rice were used at approximately 80%, 5%, and 15% (*w/w* of total grist), respectively, to match an 8 °P, low-alcohol craft lager profile. The raw materials included water, malt, wheat, rice, and hops. Hops were Czech aroma-type hops with low α-acid content (typically about 2.5–4.5%), and hopping was controlled to yield a low bitterness range of approximately 8–12 IBU, consistent with a light, low-bitter lager profile. Because raw rice requires gelatinization prior to enzymatic saccharification, a cereal-cooking step was applied. Rice was mashed together with approximately 20% high-diastatic-power malt (relative to the rice portion) and held in the rice gelatinization window of about 68–78 °C, followed by heating to a gentle boil and boiling for 20–30 min. The cooked rice mash was then returned to the main mash for saccharification. Main mashing followed a step-infusion program of 63 °C for 30 min and 70 °C for 20 min, followed by mash-out at 76–78 °C. Mash pH was controlled at 5.2–5.4. Lautering and sparging were performed at approximately 75 °C. Wort was boiled with hops and then cooled to the pitching temperature. For fermentation, a bottom-fermenting lager yeast (*Saccharomyces pastorianus*) was inoculated at approximately 10 °C. The pitching rate was controlled at 1.5–2.0 × 10^6^ cells/mL/°P, corresponding to about 12–16 × 10^6^ cells/mL for 8 °P wort. Dissolved oxygen in cooled wort was adjusted to a target of 8–10 mg/L prior to inoculation. When fermentation approached the end point (approximately 2 °P above terminal extract), temperature was raised to about 16–18 °C for a diacetyl rest. Beer was then cooled at about 1 °C/day to 0–2 °C and matured at 0–2 °C.

After collection, the samples were immediately stored at 0 °C. Sampling time was recorded using 24 h as 1 d. The main solvents and additives included ACN (≥99%, chromatographic grade) and FA (≥99%, chromatographic grade). Both were purchased from Beijing InnoChem Science and Technology Co., Ltd., Beijing, China. Ultrapure water was used. Semi-quantitative analysis was performed using a synthetic umami peptide standard PVPL (purity ≥ 95%, Nanjing Taopu Biotechnology Co., Ltd. (Nanjing, China)).

### 2.2. Instruments

Key equipment and consumables included a Milli-Q ultrapure water system (Millipore, Bedford, MA, USA) for preparing ultrapure water. Routine pipetting and volumetric preparation were performed using a 1000 μL pipette (Sinopharm Chemical Reagent Beijing Co., Ltd., Beijing, China) and 10 mL and 100 mL volumetric flasks (Weiye Metrology and Technology Research Group Co., Ltd., Beijing, China). Consumables for sample transfer and instrumental analysis included 2 mL vials (Beijing InnoChem Science and Technology Co., Ltd., Beijing, China). A VM-500S vortex mixer (Qunan Scientific Instruments, Huzhou, Zhejiang, China) and an SHB-III circulating water multi-purpose vacuum pump (Zhengzhou Greatwall Scientific Industrial and Trade Co., Ltd., Zhengzhou, Henan, China) were used for mixing and pretreatment. Sample clarification and low-temperature centrifugation were conducted using a Fresco 17 refrigerated microcentrifuge (Thermo Scientific, Waltham, MA, USA). The analytical platform consisted of a Waters UHPLC system (Waters, USA, Milford, MA, USA) coupled to a SCIEX TripleTOF 5600 series QTOF high-resolution mass spectrometer (SCIEX, Framingham, MA, USA).

### 2.3. Experimental Procedures

#### 2.3.1. Sample Preparation

A 400 μL aliquot of sample was placed in a 1.5 mL centrifuge tube and vortexed for 30 s. The sample was centrifuged at 17,000× *g* for 15 min at 20 °C. The clarified supernatant was collected and transferred to a new tube. Disturbance of the bottom pellet was avoided. The supernatant was concentrated to dryness in a vacuum centrifuge concentrator at 35 °C. The residue was reconstituted with 100 μL of 75% methanol in water by volume. The mixture was vortexed for 60 s and then sonicated for 10 min to improve dissolution. During sonication, the water-bath temperature was controlled to avoid unnecessary sample variability caused by heating. The reconstituted solution was centrifuged again at 17,000× *g* for 10 min at 20 °C. The supernatant was transferred to a vial for subsequent instrumental analysis.

#### 2.3.2. RPLC-QTOF-MS Conditions

Peptide separation was performed under reversed-phase conditions using an ACQUITY UPLC HSS T3 column (C18, 1.8 μm, 2.1 mm × 100 mm, 100 Å, Waters, USA). The mobile phases were acidified aqueous and organic phases. Mobile phase A was 0.1% FA in water by volume. Mobile phase B was 0.1% FA in ACN by volume. The injection volume was 4 μL. The flow rate was maintained at 0.20 mL/min. The total run time was 60 min. Gradient elution was performed by changing the proportion of B. The proportion of B was held at 5% from 0 to 3 min. It was increased linearly from 5% to 10% from 3 to 5 min. It was increased to 20% from 5 to 34 min. It was increased to 40% from 34 to 50 min. It was increased to 95% from 50 to 56 min and briefly maintained at high organic conditions. It was returned to 5% B from 56.1 min and re-equilibrated until the end of the run.

MS detection was performed on a TripleTOF 5600 QTOF system controlled by Analyst TF 1.7. Data-dependent acquisition was used and was also termed IDA. In each cycle, an MS1 survey scan was acquired. Up to 10 precursor ions with intensity above 100 cps were selected for MS2 fragmentation. The MS1 scan range was *m*/*z* 100 to 1500. Collision energy was set to 30 eV. An electrospray ion source was operated in positive-ion mode. Source conditions were set as follows. GS1 was 60 psi. GS2 was 60 psi. CUR was 35 psi. The source temperature was 550 °C. The spray voltage was 5500 V.

#### 2.3.3. Qualitative Identification and Semi-Quantitative Analysis of Peptides in Beer

Qualitative peptide identification and protein-source assignment were performed in PEAKS Studio. To reflect the multi-source proteins in beer, an integrated reference-library strategy was used. Protein sequences of *Hordeum vulgare*, *Triticum aestivum*, and *Saccharomyces cerevisiae* were merged to construct the search database. Sequences were obtained from the UniProt reference proteome FASTA downloads. Because peptides in beer often underwent irregular cleavage by endogenous or microbial proteases, the enzyme setting was defined as Enzyme None and matching was performed under a nonspecific digestion mode. A de novo module was enabled to improve identification of peptides not present in the database, low-coverage regions, and variant sequences. De novo tag information was included in subsequent database matching to improve sensitivity and robustness [36,37,38,39]. Mass accuracy thresholds were set to 10 ppm for precursor ions and 0.03 Da for fragment ions.

Common structural changes introduced by sample handling and the chemical environment were considered, while limiting false matches caused by an expanded search space. Variable modifications included methionine oxidation, protein N-terminus acetylation, N-terminal pyroglutamylation of glutamine or glutamate, deamidation of asparagine or glutamine, and cysteine modification associated with reduction treatment. False positives were controlled using a target-decoy strategy. A 1% FDR filter was applied at the peptide-spectrum match and protein levels [40,41,42,43].

The semi-quantitative calibrant was the synthetic PVPL peptide. The standard curve was Y = 0.0039X + 6.6831 R^2^ = 0.9998.

#### 2.3.4. Efficient Screening of Potential Umami Peptides Using Machine Learning

To more efficiently identify potential umami peptides from large-scale candidate sequences, sequence-based machine learning prediction was used as a prescreening step. The peptide FASTA format converter is provided in Supplementary File S1. Candidate peptide sequences were submitted to the prediction modules of the UMPred-FRL (http://pmlabstack.pythonanywhere.com/UMPred-FRL, accessed on 1 January 2026) online server and the TastePeptides-Meta (http://tastepeptides-meta.com/TPDM, accessed on 1 January 2026) platform. The classification outputs and prediction scores returned by each model were archived.

UMPred-FRL was a sequence-driven machine learning ensemble predictor. It output a predicted probability score from 0 to 1 and used 0.5 as the default classification threshold. A binary result of umami or non-umami was provided. The probability scores were recorded and were used to prioritize sequences to reduce the cost of downstream blind screening [44].

TastePeptides-Meta was an integrated online platform that combined a taste-peptide database with machine learning prediction. It supported search, download, and prediction, and it was used for rapid screening of taste-peptide candidates. The predicted labels and score information was recorded for cross-reference. Because training sets and feature spaces differed across models, a high score from a single model did not necessarily indicate true umami activity. A dual-model consensus strategy was adopted. Sequences classified as umami by both platforms were defined as high-confidence candidates. Candidates were then ranked and stratified based on prediction scores for subsequent quantitative validation and mechanistic analysis [45].

#### 2.3.5. Molecular Docking

Based on homology, the reference sequence of the metabotropic glutamate receptor mGluR1 (GRM1) was obtained from UniProtKB and was used as the starting point for 3D model construction. The crystal structure with RCSB ID 1EWK provided experimentally resolved coordinates for the extracellular ligand-binding domain and served as a template. The target sequence was aligned with the template on the SWISS-MODEL server to build a 3D model that included the binding pocket and adjacent secondary structures. Conservation between the template and target, as well as the distribution of insertion and deletion segments, was considered to reduce modeling artifacts while maintaining structural plausibility. After model generation, residue φ and ψ dihedral-angle distributions were evaluated using a Ramachandran plot on the SAVES v6 platform. Stereochemical plausibility was assessed based on whether backbone conformations fell within allowed regions. This assessment served as quality control before docking [46,47,48].

The receptor model was then loaded in Maestro 14.5 (Schrödinger, LLC, New York, NY, USA) and processed using a standardized preparation workflow. Protein Preparation Wizard was used to add hydrogen atoms, standardize bond orders and valences, adjust the hydrogen-bond network, and perform restrained minimization under the OPLS4 force field to relieve potential geometric conflicts. Solvent molecules and cofactors unrelated to the binding cavity were removed. Necessary lipids or ions were retained when the overall receptor conformation was not disrupted, to respect membrane-environment effects on pocket shape. LigPrep and Epik were used to generate protonation states of candidate conformations. The solution pH was set to 7.0 ± 2.0 to capture major protonation isomers and tautomers. This step unified 3D configurations and removed unreasonable charge states [49,50,51].

The grid box was defined to cover the pocket and nearby channels that could be contacted by ligands, to account for cation–aromatic interactions and hydrophobic coordination modes. Docking was performed using the Glide small-molecule workflow in standard precision (SP) mode. Post-docking minimization was allowed. Multiple low-energy conformations were retained. A more stringent XP mode could be applied for rechecking when needed. Final poses were clustered by heavy-atom RMSD, and representative poses were selected for visualization and interaction analysis, including hydrogen bonds, salt bridges, cation–aromatic interactions, and hydrophobic or van der Waals contacts [52,53,54,55,56].

#### 2.3.6. MM-GBSA Binding Free Energy Calculation

Binding free energy of the T1R1/3–peptide complex was estimated using the Prime MM-GBSA workflow in Maestro 14.5. This approach combined molecular mechanics with an implicit solvent model. Local geometry optimization was applied to the docked static complex, and energy terms were calculated along a single trajectory under consistent implicit solvent settings. A restrained minimization was first applied to buffer geometric inconsistencies introduced by docking. Potential energies of the complex, receptor, and ligand were then calculated. Binding free energy was estimated using ΔG_bind_ = G_complex_ − G_receptor_ − G_ligand_. MM-GBSA combined molecular mechanics internal energy, generalized Born polar solvation, and a surface-based nonpolar term. These contributions were decomposed into components such as van der Waals and Coulomb interactions and solvation effects to evaluate affinity trends from multiple perspectives [57,58,59,60].

#### 2.3.7. Sensory Evaluation

To ensure consistent handling conditions across samples, all samples were ad-justed to 10 mL and were presented in odor-free, covered plastic cups coded with randomized 3-digit numbers to maintain blinding. Serving and evaluation were performed in individual sensory booths under controlled room conditions (neutral lighting, minimal noise and odor interference), following general test-room guidance for sensory analysis. Samples were served at 10 ± 1 °C, and each evaluation was completed within a fixed time window after pouring to minimize carbonation-driven variability; panelists were instructed to expectorate after tasting and to rinse with room-temperature water between samples, with a 60–90 s rest to reduce carryover. A trained panel (*n* = 10; 5 male and 5 female; 20–35 years) was used. Panelists were screened for normal taste function and availability, and training was conducted according to ISO guidance for the selection and training of sensory assessors. Training included 3 sessions (1 h each) to calibrate umami intensity using reference solutions of monosodium glutamate (MSG; 0.08, 0.34, and 1.00 g/L) and to align the use of the umami scale and palate-cleansing procedure.

Four sampling time points were designed based on fermentation dynamics and corresponded to wort fermentation day 0, day 1, day 3, and day 9. At each time point, 10 independent samples were randomly taken from different fermentation tanks. Samples were coded as 1-1 to 1-10, 2-1 to 2-10, 3-1 to 3-10, and 4-1 to 4-10. This coding supported blind evaluation and data tracking. The primary focus was umami perception. This taste was mainly derived from synergistic activation of taste receptors by amino acids and 5′-nucleotides. It was associated with enhanced taste richness and overall mouthfeel fullness and was distinct from simple salty or sweet sensations. Umami intensity was rated on a 0–10 unstructured line scale anchored at 0 (not perceptible) and 9 (extremely strong). Sample order was balanced across panelists using a randomized complete-block arrangement, and each sample was evaluated in duplicate across 2 sessions on separate days to support repeatability assessment. Panel performance consistency was monitored by within-panel repeatability checks as recommended for quantitative sensory panels. Sensory data were summarized as mean ± SD. Differences among brewing nodes were tested by one-way ANOVA with Tukey’s post hoc comparisons. For peptide-spiking confirmation, a two-way mixed-effects model was applied with time point and peptide as fixed effects and panelist as a random effect. Normality and variance homogeneity were checked before parametric testing. Statistical significance was set at *p* < 0.05.

Building on the validation of the effects of umami peptides on lager beer umami by integrating chemometrics with sensory omics, six representative umami peptides (IREDIAGVVVF, ATLIDPKRGHVG, YVVIPSTE, REDIT, TDHP, and EEY) were each individually added to four brewing-stage beer samples to confirm their impacts on lager beer umami perception. Samples collected on days 0, 1, 3, and 9 of wort fermentation were coded as A, B, C, and D, respectively, and the peptide-spiked samples were coded as A-1 to A-6, B-1 to B-6, C-1 to C-6, and D-1 to D-6. The peptides were added in the following order: IREDIAGVVVF, ATLIDPKRGHVG, YVVIPSTE, REDIT, TDHP, and EEY.

The sensory scoring system followed an intensity scale commonly used in taste research. Clear and comparable levels were assigned to umami intensity to convert subjective perception into quantitative data. Scores ranged from 0 to 9 and represented a gradual increase from extremely weak to very strong. A score of 0 indicated a thin taste with limited fullness. Scores of 1 to 2 indicated a slight thinness. Scores of 3 to 4 indicated a mild increase in umami. Scores of 5 to 6 indicated moderate roundness with limited umami prominence. Scores of 7 to 8 indicated a clear improvement in mouthfeel by umami without masking crispness. A score of 9 indicated very rich and clean taste with strong mouthfilling while retaining a crisp layered profile.

#### 2.3.8. Statistical Analysis

Statistical analysis was performed mainly in the R environment using version 4.5.x and associated packages for modeling and inference. Mixed-effects models were fitted using lme4. Estimated marginal means and post hoc comparisons were obtained using emmeans. Hypothesis testing and regression scripts were cross-checked in Python 3.13.12 to improve consistency and transparency. To cover different software capabilities, one-way ANOVA was conducted in IBM SPSS Statistics 24 (Northridge, CA, USA). Adjusted p values after multiple-comparison correction were reported. The significance threshold was set at *p* < 0.05. The correlation threshold was set at r > 0.8. Data visualization and curve fitting were conducted in GraphPad Prism 10.6.1 (GraphPad Software, San Diego, CA, USA) and OriginPro 2021 (OriginLab, Northampton, MA, USA). More complex multidimensional graphics, including heatmaps and clustering plots, were generated using TBtools v2.303 (South China Agricultural University, Guangzhou, China).

## 3. Results and Analysis

### 3.1. Qualitative Identification of Peptides Across the Full Lager Beer Process and Analysis of Potential Contributions to Umami Flavor

#### 3.1.1. Predictive Analysis of Umami Peptides in Lager Beer Brewing on Day 0

In this study, peptides obtained from database searching were filtered using −10logP ≥ 15 as the confidence threshold. For de novo results, only sequences with ALC ≥ 90% were retained to ensure sequence reliability. A total of 25,592 peptides were identified. UMPred-FRL and TastePeptides-Meta were then applied to predict umami-related properties using a threshold of 0.5. A total of 7255 potential umami peptides were obtained. Qualitative and semi-quantitative peptide metrics and umami prediction results for lager beer samples on wort fermentation day 0, day 1, day 3, and day 9 are provided in Supplementary File S2. Details are included in the compressed Supplementary File S2. The 40 files corresponded to the 40 samples. Qualitative and semi-quantitative umami peptides were reported for each sample. Files were named sequentially as 1-1 to 1-10, 2-1 to 2-10, 3-1 to 3-10, and 4-1 to 4-10.

As shown in Supplementary File S2, the day 0 wort essentially represented a protein and peptide library formed after mashing and boiling. In total, 7818 peptides were identified at day 0, providing a quantitative baseline for the mash/boil-derived peptide pool prior to yeast-driven turnover. Secondary proteolysis and intracellular or extracellular peptide turnover driven by yeast growth had not yet occurred. Therefore, umami peptide sequences resembled products generated by progressive proteolysis of malt during malting and mashing. Sources were mainly barley storage proteins and associated proteins. Previous work indicated that a considerable proportion of cereal proteins could be degraded during mashing and enter the wort phase. Subsequent boiling could induce aggregation or removal. Multiple cereal proteins and their degraded peptides could still be detected in wort. Many noncanonical cleavage sites were also observed, indicating substantial processing-driven degradation. This pattern explained the coexistence of very short peptides and long fragments with clear storage-protein repeat features in Supplementary File S2.

In terms of sequence forms, the predicted umami peptides showed strong polarization. Many short peptides with 2 to 4 residues were present, such as AE, EV, EA, DGV, and DKP. This pattern indicated dense terminal trimming and exopeptidase modification in wort. These short peptides were more likely to represent a nitrogen pool and a reactive substrate pool. They were not necessarily equivalent to perceptible umami contribution. Meanwhile, many fragments with lengths far exceeding 15 residues were also observed. These included QQ series and QPQP repeat fragments rich in Q and P, as well as long peptides with clear hydrophobic segments. Based on experience summarized from sensory databases, classic umami peptides were often within 2 to 15 amino acids. Therefore, longer sequences were flagged primarily because internal motifs within these sequences matched umami-associated patterns used by the prediction models. This was consistent with prediction strategies that rely on fragment matching or motif occurrence. In such cases, labeling a long peptide as umami-related suggested potential enrichment of active motifs. It did not directly imply that the full-length peptide produced the same umami intensity in the oral cavity.

At the amino-acid composition level, umami peptides in the day 0 sample showed a pronounced fingerprint of malt storage proteins. Small residues such as A, G, and V occurred frequently. This tendency supported compact sequences with low steric hindrance. It was consistent with fragments that could remain detectable after thermal processing of cereal proteins. Dense enrichment of Q and P was also notable. Many motifs such as QQQ, QPQQ, QPQPQQ, and PQQPQQ were present. These motifs closely matched repeat motifs of barley hordein, especially C-hordein. Literature commonly summarized the repeat regions using motifs such as PQQPFPQQ or related repeats. These Q and P rich fragments were not necessarily the most typical umami framework. They could markedly affect solubility, aggregation tendency, and continuity of mouthfeel in the peptide pool. They also provided substrates for further cleavage during subsequent fermentation stages. E and D also recurred across many sequences. The presence of negatively charged residues was compatible with umami receptor recognition. Human umami perception was mainly mediated by glutamate-related receptor pathways such as the T1R1/T1R3 heterodimer. These pathways were generally more sensitive to ligands bearing acidic side chains. Therefore, umami at this stage was more likely to appear as a background support dominated by glutamate-related amino acids and acidic short peptides. It was less likely to show the clearer umami elevation observed in later fermentation.

When samples 1-1 to 1-10 were viewed as subsets within the same time point, a stable core sequence pool repeatedly appeared. Examples included AERGSY, AGEQAFHRGGV and its extensions, VEVPGGLTVA, GGTIVNS, PVVETTAEAAAGDAKPA, and fragments related to LAIDTRVG or IDTRVGV. Repetition across subsets suggested high abundance of the parent proteins and relatively fixed cleavage routes. In contrast, cysteine-enriched fragments appearing in a few subsets and longer peptides with clear hydrophobic segments were consistent with heat-stable, disulfide-rich barley proteins that persisted in wort. Such sources included lipid transfer proteins and α-amylase/trypsin inhibitor families that have been reported as abundant in wort. Such sources could include associated proteins or enzyme inhibitor proteins. Wort proteome studies also indicated that LTP1 and alpha amylase trypsin inhibitor proteins were relatively abundant in wort. If these fragments were predicted as umami peptides, the contribution was more likely indirect. Such effects may arise through carrying acidic short motifs or providing thickness and persistence in mouthfeel. These effects could indirectly amplify perceptible umami.

Overall, the umami peptide distribution in day 0 wort resembled a multilayer structure. Short peptides formed the basic pool. Medium-length peptides of 6 to 15 residues were more likely to represent the main potential umami contributors. Hordein-like long fragments rich in Q and P provided a background framework.

#### 3.1.2. Predictive Analysis of Umami Peptides in Lager Beer Brewing on Day 1

As shown in Supplementary File S2, peptides predicted to be umami-related in the day 1 sample displayed markedly higher complexity and strong heterogeneity. In total, 9275 peptides were identified on day 1, providing a concise quantitative anchor for this time point and reflecting the rapid expansion of the peptide pool at the onset of fermentation. The key feature was not dominance of a few single sequences. Multiple structural families coexisted within the same time window. Sequence lengths ranged from dipeptides to peptides with dozens of residues. Homologous fragments recurred across groups. This pattern indicated that these peptides were not incidental noise. A stable peptide library had been formed by sustained degradation of malt protein backbones during mashing and early fermentation. Under selection pressure from rapid yeast proliferation and nitrogen assimilation, the library was reshaped. Related studies on beer proteomics and cereal protein source analysis also indicated that detectable peptides in beer were often contributed by cereal storage proteins with a smaller contribution from yeast proteins. Processing could markedly alter detectable lineages and relative abundance.

From a taste chemistry perspective, the very high proportion of short peptides was a major feature of the day 1 distribution. Many dipeptides and tripeptides were present. Many oligomeric fragments were dominated by small residues. This pattern suggested strong combined action of exopeptidases and endopeptidases. Proteins were rapidly cleaved into forms more amenable to migration and transport. Enrichment of acidic residues was also clear. E and D appeared frequently in short peptides. They formed highly repeated sequence patterns together with small residues such as A, G, and V. This pattern aligned with structural tendencies reported for umami-related peptides from multiple sources. Umami-associated peptides tended to be short and tended to carry negatively charged side chains such as glutamate or aspartate. These features supported direct umami perception or a mouthfeel thickening effect with umami as the base.

Alongside these rapidly assimilable short peptide families, many Q and P rich repeat families were also present. Examples included QPQQ, QQCCQQLPQIP and its extensions, and longer fragments such as QPQQQPQ. These sequences closely matched typical features of barley prolamin storage proteins. P and Q proportions were high. Protease resistance was often strong. Therefore, these fragments were more likely to persist as residues after brewing and early fermentation. Mass spectrometry studies on barley prolamin peptides in beer also showed clear P and Q enrichment and repeatable spectral fingerprints across beer samples. A critical point should be noted. Model classification of these P and Q rich peptides as umami-related did not necessarily mean that contribution was driven by canonical umami receptor activation. More plausible mechanisms included effects on viscosity, interactions with polyphenols that altered colloidal structure, or binding with other taste-active molecules. These pathways could indirectly shape the somatosensory background of umami. Therefore, the day 1 distribution reflected not only enhancement of an umami signal. It more likely reflected coupled formation of umami signals and colloidal mouthfeel background.

Another prominent group included cysteine-rich sequences and their extended series. Examples included LVAPGQCNLATIHNVRYCPAVE and longer variants, as well as multiple Cys cluster related fragments. Beer proteomics studies generally suggested that barley disulfide-rich proteins had high thermal stability and process tolerance. They remained detectable during early fermentation. They have been linked to foam and mouthfeel structure through hydrophobic interfacial interactions. Day 1 usually corresponded to an active phase of primary fermentation. Yeast nitrogen demand and uptake intensity were high. Small peptides and amino acids tended to be transported and assimilated first. In contrast, longer fragments rich in P and Q were more likely to persist due to transport limitations and protease resistance. The detected umami-related peptide spectrum thus reflected a cross-sectional outcome after simultaneous generation and consumption. It included nutrition-type short peptides undergoing rapid metabolic throughput. It also included tolerant peptides that were difficult to fully remove in the early fermentation environment. Studies on oligopeptide transport systems in fungi and yeast also showed that transporters had selectivity for substrate length and composition. This selectivity directly altered the structural distribution of peptides that accumulated. It supported a pattern in early fermentation that combined short peptide enrichment with persistence of specific difficult-to-degrade fragments.

In summary, the day 1 umami peptide distribution showed a clear two-layer structure. The first layer was an assimilable short-peptide pool enriched in acidic residues. It was more likely to carry the main contribution to direct umami and umami enhancement. The second layer was a tolerant residual peptide library represented by P and Q repeat fragments and cysteine-rich framework fragments. This layer more likely supported umami through mouthfeel structure and interaction networks.

#### 3.1.3. Predictive Analysis of Umami Peptides in Lager Beer Brewing on Day 3

As shown in Supplementary File S2, peptides in the day 3 sample showed clear length stratification and residue preference. In total, 8836 peptides were identified on day 3, providing a single quantitative anchor for this time point within the large peptidomics dataset while contextualizing the observed compositional shifts. The main body consisted of short peptides of 2 to 10 amino acids. A small number of medium to long fragments were also present. This pattern suggested ongoing supply from protein degradation and selective retention of some fragments. The sequences were enriched in small residues such as A, G, V, and T. Many acidic residues such as D and E were also present. In beer peptidomics, this combination was often interpreted as a barley-derived peptide library formed after multipoint cleavage of malt proteins under processing and fermentation conditions.

Two highly indicative structural fragment types were observed. One type included repeated Q and P rich fragments. Many sequence clusters were centered on motifs such as QPQQ and QQQ. These patterns matched characteristics of proline-rich and glutamine-rich peptides generated after degradation of cereal storage proteins. Such peptides were difficult to mineralize completely during fermentation. They could accumulate in beer and participate in colloidal behavior. Another type included fragments with multiple cysteine sites and their extended sequences. Longer fragments containing Cys sites were present. Their features matched peptides derived from heat-tolerant barley proteins commonly detected in beer. Such proteins were often linked to foam and interfacial stability. They were retained under process conditions rather than fully removed.

When structural signals related to umami potential were emphasized, the most notable feature was frequent occupancy of D and E among many short peptides. Sequences with consecutive acidic residues recurred, such as AAADDEEMKL, DEEKFP, and FVDDQ. At the mechanistic level, characterization of the umami receptor T1R1/T1R3 supported receptor binding and conformational activation as a plausible route for contributions from certain taste peptides. Structure–activity studies of taste peptides also indicated that introducing acidic residues into short peptides was often associated with umami or umami enhancement. This association was stronger when acidic sites co-occurred with a proportion of hydrophobic residues. Such combinations could form spatial and charge distributions recognizable by the receptor. Therefore, the day 3 distribution did not indicate a few highly homologous classic sequences. It instead suggested a diverse and redundant community of candidates. Acidic short peptides formed the core. Multiple short peptides dominated by small residues provided a broader candidate background.

From mid to late fermentation, yeast nitrogen uptake showed priority and pathway selection. Free amino acids and some transportable short peptides were continuously consumed. Meanwhile, ongoing cereal protein degradation continued to input new peptides. A dynamic balance was formed. Yeast possessed multiple peptide transport and utilization capacities. Oligopeptides within certain length ranges could be taken up and incorporated into nitrogen metabolism. Sequence properties affected uptake probability and further degradation. This mechanism explained coexistence of many short peptides and specific enriched sequences on day 3. The pattern more likely reflected combined effects of continuous generation and selective removal. It did not reflect a static snapshot from a single source event.

#### 3.1.4. Predictive Analysis of Umami Peptides in Lager Beer Brewing on Day 9

As shown in Supplementary File S2, the day 9 umami peptide spectrum showed clear late fermentation characteristics. In total, 8975 peptides were identified in the day 9 sample, providing a single quantitative anchor for this terminal time point within the large-scale peptidomics dataset. A multi-source peptide library with broad length coverage supported potential umami driving. Dominance by a few single superior sequences was not observed. Based on nitrogen metabolism rules in beer, free amino nitrogen and utilizable small peptides in wort were continuously assimilated as fermentation progressed. Peptides retained at the late stage were more likely to originate from residual fragments that were difficult to further utilize. They could also originate from supplementation driven by late-stage cell turnover and protein degradation. These inputs could sustain a high-complexity peptide collection at the terminal node.

In terms of sequence distribution, lists from 4-1 to 4-10 consistently showed strong length stratification. Many very short peptides appeared, such as AC, CH, DP, DQ, EA, and EE. This pattern suggested frequent low-oligomer fragments and free termini. Stable medium and short peptide frameworks were also present. Examples included AADALLLKVN, ALDTRVGV, ELSESEMR, and EPQQQVPVEVMR. These recurred across subsets. This pattern indicated a core set of peptide signals that remained detectable at the late stage. Longer peptides were also present, such as AAAAFPGFGTTGSADAQKR and multiple fragments with potential multi-site modifications. This pattern indicated that the late node did not represent a fully hydrolyzed end state. It reflected a dense background of short peptides combined with ongoing supply and persistence of soluble protein fragments.

In compositional terms, the late-stage umami peptide spectrum could be summarized as several complementary clusters. One cluster consisted of acidic residue enriched short peptides. E and D occurred frequently and were often present as dipeptides or tripeptides. These peptides carried clear negative charge and high aqueous exposure. They were more likely to directly contribute to umami or umami enhancement signals. A second cluster consisted of Q and P rich repeat fragments. Many QPQPQ, QPQQ, and EPQQ related fragments were present. These peptides typically showed high conformational flexibility and low protease accessibility. They resembled reserve-like residual fragments that were difficult to fully remove at the late stage. Their direct driving force for umami could be weaker than that of acidic short peptides. They could amplify aftertaste through effects on colloidal stability and oral retention. A third cluster consisted of cysteine-containing fragments. Structural constraints and potential disulfide related behavior were implied. This cluster was consistent with proteins associated with beer foam and interfacial activity. Model classification of these fragments as umami peptides did not necessarily reflect their true sensory contribution. It instead suggested a need to be cautious about systematic overestimation by prediction models for stable protein fragments.

In terms of protein sources, the late-stage spectrum was consistent with established beer proteomics conclusions. Soluble beer proteins and their fragments mainly originated from barley raw material proteins and yeast proteins. Many protein cleavage events and peptide heterogeneity were observed in finished beer. Nonspecific cleavage and processing modifications could markedly increase the number of detectable peptides and shape the complex terminal spectrum. This mechanism explained the coexistence of many short peptides and retained longer fragments on day 9. The former corresponded to high-turnover degradation products. The latter corresponded to framework fragments that were more stable in the beer matrix.

Overall, at wort fermentation day 0, umami peptide clusters mainly inherited products from malt extraction and proteolysis during mashing. Sources were dominated by barley storage proteins and their fragments. Because barley prolamin families were generally rich in Gln and Pro, the wort ends often carried a QP enriched peptide framework. Such sequences increased structural redundancy of the peptide pool. They also provided nitrogen components and potential umami frameworks that were more difficult to rapidly remove during subsequent fermentation.

At day 1, yeast was within the most active window for proliferation and assimilation. Assimilable amino acids and some small peptides served as assimilable nitrogen. Studies indicated that yeast could consume 20% to 40% of wort peptide nitrogen. Transmembrane uptake tended to favor oligopeptides within 3 amino acids. Therefore, overall abundance and sequence diversity of umami peptides were likely to contract rapidly. Residual fractions tended to shift toward longer chains or Pro rich sequences. Pro assimilation was limited under anaerobic fermentation conditions. This limitation further increased relative retention of Pro enriched fragments. The peptide pool thus shifted from diversified input toward selective enrichment of tolerant sequences.

By day 3, primary fermentation maintained high nitrogen demand. The peptide pool continued to undergo passive filtering. Uptake-eligible small peptides were continuously depleted. Pro tolerant QP fragments and fragments from thermally stable barley proteins increased in proportion. Umami contribution was more likely carried by a smaller set of short peptides rich in Glu or Asp. The key factor was that negatively charged side chains provided more direct ligand information to the umami receptor pathway. The contribution was less driven by nitrogen source meaning.

By day 9, fermentation rate declined and uptake pressure weakened. Residual umami peptides more directly reflected combined outcomes of solubility and tolerance. A mild replenishment effect from cell turnover could also be superimposed. Terminal samples thus showed a pattern dominated by small peptides with highly dispersed sequences. Acidic residues and QP frameworks coexisted. Overall, umami peptide succession from day 0 to day 9 could be summarized as high-complexity input, rapid assimilation-driven removal in early fermentation, selective retention in mid fermentation, and stabilization toward the terminal stage. This process shaped the final perceptible umami.

### 3.2. Mechanistic Analysis of Interactions Between Umami Peptides and the Receptor T1R1/T1R3

**Figure 1:**
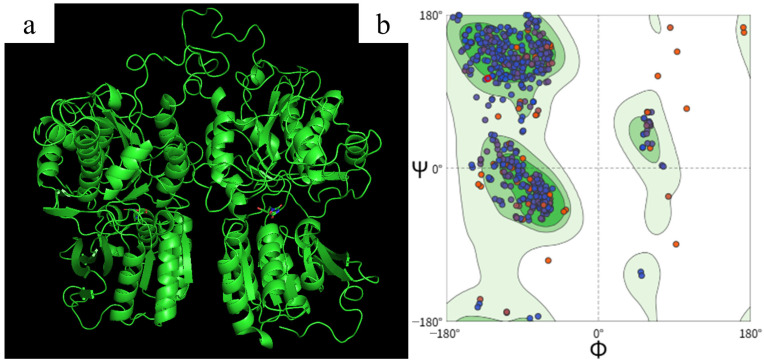
Homology model and evaluation of the umami receptor T1R1/T1R3. (**a**) Structural model of the T1R1/T1R3 heterodimer. (**b**) Ramachandran plot of the model structure.

On this basis, 145 representative umami peptides were selected for molecular docking according to predicted umami scores and related studies. T1R1/T1R3 is a type I taste receptor heterodimer that mediated umami perception [61,62,63,64,65,66]. It belongs to the class C GPCR superfamily. Its extracellular ligand-binding region exhibits a typical Venus flytrap (VFT) fold. Signal transmission is completed in coordination with the cysteine-rich region and the transmembrane region. To reduce modeling complexity and facilitate comparison of subunit contributions to ligand recognition, the heterodimer was separated into the T1R1 and T1R3 subunits for homology modeling. A Ramachandran plot was used to examine backbone dihedral-angle distributions and to screen for pronounced conformational abnormalities. As shown in Figure 1a, the resulting conformations showed a relatively closed T1R1 and a more open T1R3. This spatial relationship formed a cavity geometry near the dimer interface that could accommodate or allow adhesion of longer peptides. This conformational feature was consistent with prior understanding of VFT conformational differences in T1R1/T1R3 and their effects on ligand accommodation. Previous studies suggested that ligand recognition in this receptor was not determined by a single residue cluster. Multiple sites within the VFT and near the interface could jointly contribute to ligand anchoring and conformational stabilization. In addition to free amino acids, umami peptides derived from food protein hydrolysis were also proposed to interact with T1R1/T1R3. This proposal provided a plausible receptor basis for binding modes of longer peptides [67,68].

The reliability of homology modeling was closely associated with template homology. In general, when sequence identity between target and template exceeded 30%, sequence identity served as an effective predictor of model accuracy. Strict structural quality control and subsequent computational validation were still required. Sequence identities of 20% to 35% were often considered the twilight zone for alignment. Models in this range were more susceptible to alignment errors and local structural uncertainty. In this study, sequence identities for T1R1 and T1R3 were 34.34% and 33.55%, respectively. This range supported homology modeling but required strengthened quality control. Ramachandran statistics (Figure 1b) showed that 97.7% of residues fell within allowed regions. Among them, 87.7% were in the most favored region and 10.0% were in additionally allowed regions. The generously allowed region accounted for 1.8%, and the disallowed region was below 0.5%. Overall, the model met basic requirements at the backbone geometry level for docking and mechanistic analysis [67,69,70,71]. It could serve as a structural basis for subsequent screening of ligand-binding poses and interpretation of interactions.

Semi-flexible docking was performed using the Schrödinger 14.5 docking suite. Other parameters were kept at default settings, and only the pose with the lowest docking score was retained. All 145 umami peptides were successfully docked (Table 1). Peptide length ranged from 2 to 20 amino acid residues. A pattern of short-peptide enrichment with a long-peptide tail was observed. Specifically, 7 dipeptides, 37 tripeptides, 41 tetrapeptides, 30 pentapeptides, 8 hexapeptides, 4 heptapeptides, 2 octapeptides, 3 decapeptides, 2 11-mers, 3 12-mers, 2 13-mers, 1 14-mer, 2 15-mers, 2 17-mers, and 1 20-mer were included. Peptides of 3 to 5 residues totaled 108 and accounted for 74.48%, indicating that umami peptides were closer to a soluble short-peptide pool formed after stepwise protein hydrolysis in the fermentation system. In terms of amino acid features, 107 peptides contained E or D and accounted for 73.79%. These acidic sites provided a structural basis for forming salt bridges and hydrogen bonds with positively charged or polar residues in the receptor pocket. This pattern also agreed with the commonly summarized feature of acidic anchoring in umami peptides.

Docking results showed that the docking scores of these 145 peptides ranged from −12.568 to −3.269. More negative values typically indicated more favorable predicted binding, but this metric was more suitable for ranking and relative comparison than for direct equivalence to sensory intensity. Notably, peptide length showed a significant negative correlation with docking score, with a correlation coefficient of approximately −0.70. This relationship reflected a general rule in which increased length increased contact area. After grouping by peptide length, the mean docking score was −6.499 for 2 to 5 peptides, −8.029 for 6 to 9 peptides, and −9.680 for peptides of length 10 and above. This gradient also suggested that longer peptides more readily achieved greater geometric complementarity with the pocket.

When the interaction score was decomposed into Glide ecoul and Glide evdw, the dominant terms differed between short and long peptides. For 2 to 5 peptides, the mean ecoul was approximately −26.183 and the mean evdw was approximately −25.512. Electrostatic interactions contributed a larger proportion and often depended on a few key salt bridges and hydrogen bonds for efficient anchoring. For peptides of length 10 and above, the mean ecoul was approximately −33.843 and the mean evdw was approximately −60.655. Van der Waals interactions and hydrophobic complementarity became the major contributors and reflected lower energy driven by the accumulation of multiple weak interactions. This difference was compatible with the structural feature of the VFT pocket, which could provide multiple contact sites. Longer peptides tended to spread across a larger surface within the pocket, whereas shorter peptides relied more on precise placement of charged groups at key positions within the cleft.

It should be emphasized that longer peptides did not necessarily produce stronger umami even when more favorable docking scores were obtained. One reason was that docking scores reflected binding tendency under a static pose, whereas umami intensity was closer to a kinetic outcome that depended on whether an active conformation could be induced and stabilized. Another reason was that solubility, diffusion, and matrix binding could limit the effective concentration of longer peptides at the receptor. Perceived intensity in real systems was often jointly controlled by threshold behavior and release dynamics. A third reason was that some longer peptides could favor occupancy binding or interact with T1R3 in different modes, which might not efficiently trigger signal amplification.

Based on these results and related studies, 6 peptides were selected as a representative anchor peptide set for subsequent discussion of binding modes [63,66,70,72,73,74]. The selection considered coverage of favorable docking-score ranges, coverage of the peptide-length spectrum, and differences in ecoul and evdw contribution patterns. IREDIAGVVVF (11 residues, score −11.775) and ATLIDPKRGHVG (12 residues, score −11.757) reflected mixed electrostatic characteristics with acidic sites and local basic sites. Their ecoul values were −30.600 and −33.431, respectively. These peptides were inferred to more readily form salt-bridge and hydrogen-bond networks and to achieve orientation within the cleft. YVVIPSTE (8 residues, score −11.337) was not the longest peptide, but it showed a very strong evdw (−55.182) and a lower electrostatic proportion. This peptide served as a reference for hydrophobic complementarity at an intermediate length. REDIT (5 residues, score −8.381), TDHP (4 residues, score −9.114), and EEY (3 residues, score −8.193) jointly represented the precise anchoring route for short peptides. These peptides relied on terminal carboxyl groups and polar side chains to establish a limited number of key interactions within the VFT cleft.

### 3.3. Molecular Mechanism Analysis of Six Umami Peptides and Their Receptor Proteins

**Figure 2:**
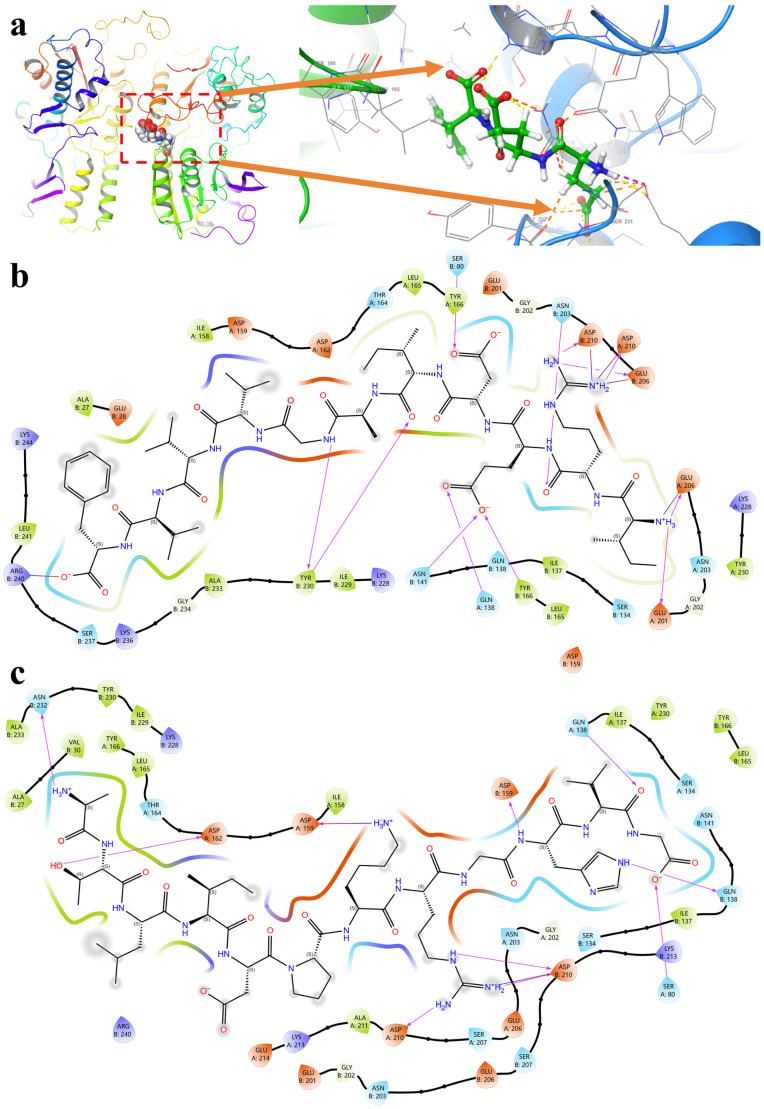

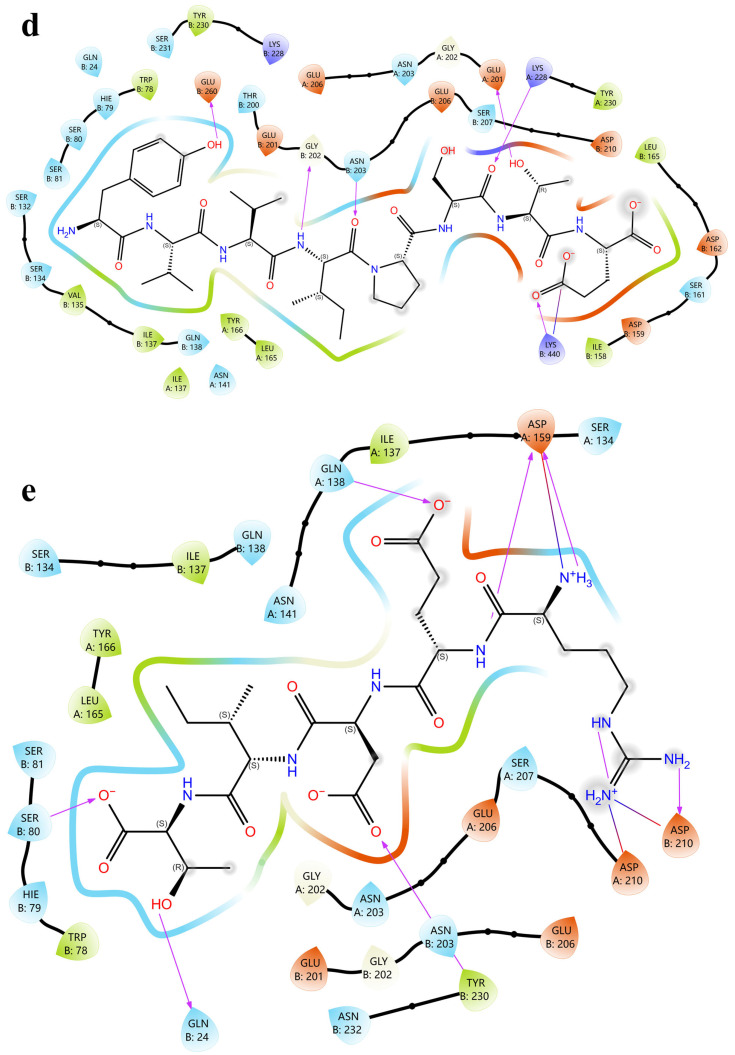

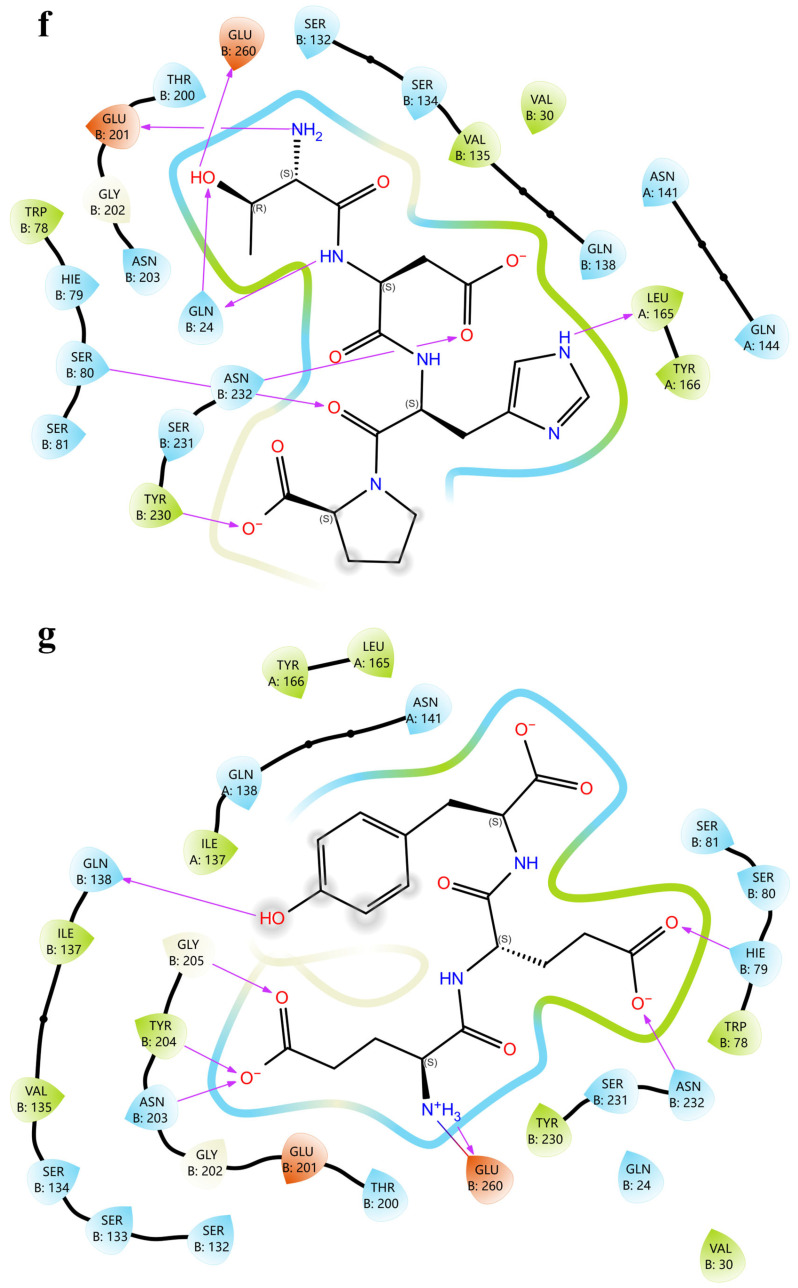
Schematic of molecular docking. Panel (**a**) showed an overall view on the left and a close-up view of interaction features on the right. Hydrogen bonds were marked in blue. Halogen bonds were marked in purple. Salt bridges were marked in red. Aromatic hydrogen bonds were marked in cyan. π–π stacking was shown in blue. π–cation interactions were shown in green. Panels (**b**–**g**) showed 2D binding diagrams. These diagrams were based on the docked T1R1/T1R3 complexes with IREDIAGVVVF, ATLIDPKRGHVG, YVVIPSTE, REDIT, TDHP, and EEY.

As shown in Figure 2a, the umami receptor T1R1/T1R3 was a heterodimer of type I taste receptors. Ligand capture was mediated by the extracellular VFT domain. Ligand-induced VFT conformational convergence transmitted signals to downstream domains. This process then triggered rearrangement of the transmembrane region and receptor activation. In the binding mode shown in Figure 2b, charged groups of peptides served as anchoring elements. Terminal carboxyl groups formed salt bridges or directional hydrogen-bond networks with basic sites on the receptor, such as Arg240 and Lys228. Acidic residues within peptides also formed multipoint hydrogen bonding and electrostatic matching with acidic or polar residues on the receptor, such as Glu206, Asp210, Asn141, and Gln138. These interactions increased the stability of the bound conformation. Meanwhile, hydrophobic side chains of Ile and Val formed hydrophobic complementarity with hydrophobic residues in the pocket, such as Leu165, Tyr166, and Ile158. The aromatic ring of Phe further contributed hydrophobicity and shape complementarity. This pattern reflected typical receptor recognition by umami peptides, in which charge pairing enabled positioning and hydrophobic packing optimized affinity.

As shown in Figure 2c, the terminal amino group at the peptide N-terminus and the hydroxyl group of Thr provided highly polar anchoring points. Directional hydrogen-bond networks were preferentially established with polar residues in the pocket. Conformational freedom of the ligand was thereby reduced. The Asp side chain and backbone carbonyls were further aligned with acidic sites such as Asp159 and Asp162. Hydrogen bonds or salt-bridge-like strong interactions could be formed. These interactions imposed more stable geometric constraints in the entrance region. The second half of the peptide was enriched in basic groups. Positive centers of Lys and Arg showed pronounced charge complementarity with Asp210, Glu206, and Glu214 and locked binding orientation. The imidazole ring of His could reinforce local stability through hydrogen bonding and aromatic-associated interactions with residues such as Gln138. Leu, Ile, and Val increased cavity matching through hydrophobic filling.

Umami peptides were mainly embedded in the VFT pocket of T1R1/T1R3 and were stabilized through multipoint weak interactions. As shown in Figure 2d, the phenolic hydroxyl of the N-terminal Tyr formed a directional hydrogen bond with Glu260. The aromatic end was thereby anchored near the pocket entrance. Hydrophobic segments preferentially matched a nonpolar microenvironment composed of Val135, Ile137, Leu165, Tyr166, and Tyr230 and provided the main hydrophobic driving force. Backbone carbonyl and amide groups in the middle of the peptide formed hydrogen-bond pairing with Gly202 and Asn203. This pairing helped extend the peptide along the pocket path and reduced conformational freedom. The carboxyl group of the C-terminal Glu showed electrostatic complementarity with positively charged sites. The schematic indicated salt bridges or charge-assisted hydrogen-bond networks with Lys440 and Lys228. Geometric constraints could also be formed with nearby acidic residues such as Glu201 and Asp210, which locked the ligand terminus in the deeper pocket region.

At the mechanistic level, REDIT was anchored in the extracellular recognition cavity through multipoint cooperativity (Figure 2e). The positively charged N-terminal amino group and the guanidinium group of Arg provided major electrostatic driving forces. Salt bridges or directional hydrogen-bond networks were formed with acidic residues. Strong interactions with Asp159 and Asp210 were shown. Additional polar constraints with Glu206 were also observed. These interactions markedly reduced conformational freedom of the ligand in the cavity. Side chains of Glu and Asp in the middle segment provided anionic sites and formed compensatory hydrogen-bond pairing with polar residues such as Gln138 and Asn203. Matching to the VFT-like cavity geometry was strengthened. Meanwhile, the hydrophobic side chain of Ile preferentially inserted into a hydrophobic fence formed by Leu165, Tyr166, Trp78, and His79. This region provided a stabilizing background of noncovalent interactions. The C-terminal Thr was anchored through hydrogen bonding between the terminal carboxyl group and Ser80 and Ser81. A supplementary polar connection could also be formed between the hydroxyl group and Gln24.

At the mechanistic level, as shown in Figure 2f, TDHP was stabilized in the VFT cavity mainly through multipoint weak interactions. The N-terminal amino group participated in interactions with the acidic site GLU201. The hydroxyl group of Thr formed a hydrogen-bond network with GLU260 and the polar residue GLN24. The entrance end of the peptide was thereby locked. The carboxyl group of the Asp side chain formed a two-point interaction with TYR230 and ASN232. This interaction provided a key negative anchoring point and reduced backbone fluctuation. The imidazole ring of His was located near the hydrophobic boundary of the pocket and matched the favorable hydrophobic and aromatic environment around LEU165 and TYR166. Nearby polar sites such as GLN138, ASN141, and GLN144 provided compensation for water bridging and orientation. Conformational rigidity of Pro further reduced freedom and improved fit to cavity curvature, which increased geometric stability of the bound pose.

As shown in Figure 2g, the VFT domain at the N-terminus maintained a dynamic balance between opening and closing. Ligand binding stabilized specific conformations and transmitted changes along the dimer interface to the transmembrane region. Downstream signal transduction was then initiated. Based on the interaction mode in the schematic, EEY relied on a carboxyl cluster for charge anchoring. The terminal carboxyl group formed continuous hydrogen-bonding and electrostatic cooperativity with GLU260, GLU201, THR200, and ASN232. Additional polar stabilization was obtained near ASN203. A backbone carbonyl formed a hydrogen bond with HIE79. The ligand maintained a stable orientation at the pocket entrance defined by TRP78, SER80, and SER81. The phenolic hydroxyl of Tyr formed a bond with GLN138. The aromatic face was compatible with TYR204. The ligand was further confined within the space enclosed by ILE137, GLN138, ASN141, LEU165, and TYR166. The tendency toward VFT closure was thereby increased, and triggering efficiency of the umami signal was strengthened.

Overall, umami peptides were preferentially embedded in the extracellular VFT recognition cavity of T1R1/T1R3. Positioning and stabilization were jointly achieved through charge pairing and hydrophobic complementarity. Terminal carboxyl groups often formed salt bridges or directional hydrogen bonds with basic sites such as Arg240, Lys228, and Lys440. Acidic side chains within peptides further established electrostatic matching and multipoint hydrogen-bond networks with Glu206, Glu201, Glu260, Asp210, Asp159, and Asp162. Polar residues in the pocket, including Asn141, Asn203, Asn232, Gln138, Gln24, Ser80, Ser81, and Thr200, provided continuous orientational constraints and water-bridge compensation, which reduced ligand conformational freedom. Meanwhile, a nonpolar microenvironment formed by Val135, Ile137, Leu165, Tyr166, Trp78, His79, Tyr230, and Tyr204 showed shape complementarity with hydrophobic side chains of peptides and contributed the main hydrophobic driving force. These coordinated multipoint weak interactions stabilized the VFT closure tendency and transmitted conformational changes along the dimer interface to the transmembrane region, which ultimately promoted receptor activation and umami signal output.

### 3.4. Analysis of MM-GBSA Binding Energy Results for Six Umami Peptides

As shown in Table 2, within the MM-GBSA end-point free energy framework, binding energy was decomposed into gas-phase interactions and solvation terms. Empirical terms for hydrogen bonding, lipophilicity, and cavity filling were also included to explain sources of stabilization in the binding pocket. In general, a more negative dG Bind (NS) indicated more favorable predicted binding. vdW and Coulomb reflected short-range dispersion and electrostatic effects. Solv GB represented polar solvation compensation or penalty. Hbond, Lipo, and Packing described directional hydrogen bonding and hydrophobic complementarity that better matched the pocket environment.

From the component structure, nonpolar contributions showed a consistent dominant role for all peptides. These contributions could be approximated by vdW + Lipo + Packing. YVVIPSTE showed the strongest nonpolar term at −180.41. IREDIAGVVVF and ATLIDPKRGHVG reached −124.74 and −128.73, respectively. These results indicated that hydrophobic complementarity and shape matching were major driving forces in this receptor system. By contrast, Coulomb + Solv GB was positive for all six peptides. Values were approximately 44.55, 70.52, 149.87, 14.35, 49.31, and 46.56. Under the sign convention of this output, electrostatic-related terms represented a net cost. This cost needed to be offset by dispersion and hydrophobic terms and by part of the hydrogen-bond term.

The advantage of IREDIAGVVVF arose from accumulation of multiple favorable components. Its vdW was −105.34. Lipo was −19.40. Hbond was −12.55. These values indicated tight hydrophobic filling with stable directional polar pairing. Coulomb was 54.12 and Solv GB was −9.57. Electrostatic cost was therefore kept within a relatively controllable range. Total energy reached −92.74. This pattern was more consistent with a binding logic in which a long peptide first achieved positioning through a hydrophobic framework within the VFT cavity, and orientation was then locked by a limited number of electrostatic sites.

ATLIDPKRGHVG also showed strong vdW at −108.80. Lipo reached −18.98 and suggested a comparable hydrophobic framework. However, Solv GB was 37.17 and imposed a pronounced unfavorable compensation. Coulomb was only 33.35, yet the net electrostatic term increased to about 70.52. Total binding energy was therefore shifted to −67.86. In physical terms, this pattern suggested a higher desolvation cost for polar groups. Electrostatic compensation gained in the pocket was insufficient to fully offset this cost. A configuration of strong hydrophobicity with a relatively large polar penalty was indicated.

YVVIPSTE showed the most representative pattern. Its vdW was −160.02 and indicated very strong short-range dispersion and shape matching. Hbond and Lipo were −10.57 and −19.28, respectively. Coulomb reached 409.43 and was largely offset by Solv GB of −259.56. The net electrostatic term remained about 149.87. Total binding energy was therefore reduced to −41.11. This peptide appeared to gain strong hydrophobic complementarity while introducing electrostatic mismatch or insufficiently screened charge distribution. Electrostatic terms became the main limiting factor.

Total energy of REDIT was −60.17. Its nonpolar term was only −68.41 and was lower than that of the longer or more hydrophobic peptides. Its net electrostatic term was about 14.35 and was the lowest among the six peptides. Electrostatic cost was therefore constrained to a small range. Packing was −1.90 and showed the largest magnitude in this set, which suggested a tighter geometric fit in the pocket. This combination was more consistent with a mode in which short peptides achieved stable orientation through charge pairing and consolidated binding through moderate hydrophobic filling.

dG Bind (NS) of TDHP and EEY were −35.37 and −33.18, respectively. Overall binding was in a weaker range. Their nonpolar terms were similar at −74.01 and −72.87. Differences mainly arose from the balance between net electrostatic cost and hydrogen bonding. Hbond of TDHP was −10.67 and exceeded that of EEY at −6.87. Directional hydrogen bonding therefore contributed more clearly to stabilization of TDHP. Net electrostatic terms were still about 49.31 and 46.56, respectively. Total binding energy therefore could not decrease further.

At the mechanistic level, stabilization of umami peptides in the VFT pocket was mainly driven by nonpolar forces. Persistent negative contributions of vdW and Lipo indicated that hydrophobic side chains obtained major binding gains within the hydrophobic microenvironment formed by Val135, Ile137, Leu165, Tyr166, Trp78, and His79. Charged residues mainly contributed to orientation and anchoring. Salt bridges or hydrogen-bond networks were formed with Lys228, Arg240, Glu206, Asp210, Asn141, and Gln138. Ligand conformational freedom was thereby reduced. Meanwhile, net electrostatic terms were costs for all six peptides. This pattern suggested that desolvation and insufficient electrostatic matching could often limit further optimization of binding energy. Therefore, a higher docking score or a longer peptide chain did not necessarily correspond to stronger umami output. Receptor activation also depended on how efficiently the binding pose promoted VFT closure and downstream conformational transmission.

### 3.5. Confirmatory Analysis of the Effects of Representative Umami Peptides on Lager Beer Umami

**Figure 3:**
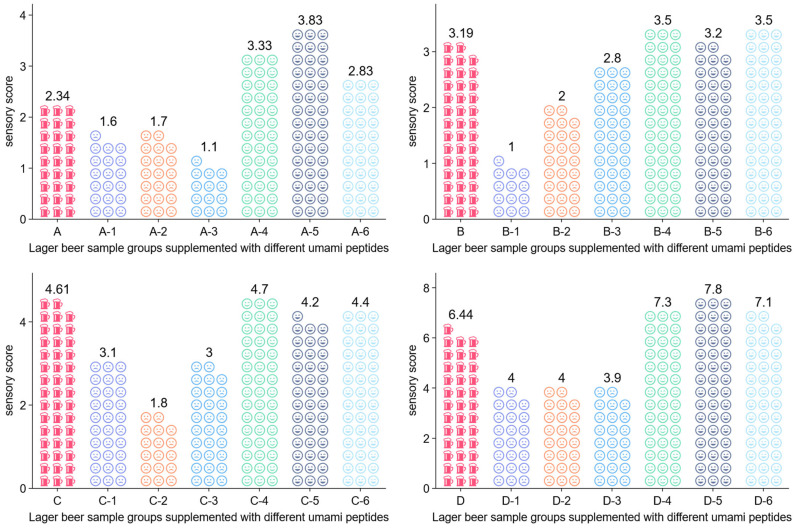
Comparison of umami perception in lager beer before and after the addition of representative umami peptides. Note: Wort fermentation samples collected on days 0, 1, 3, and 9 were coded as A, B, C, and D, respectively. The peptide-spiked samples were coded as A-1 to A-6, B-1 to B-6, C-1 to C-6, and D-1 to D-6. The peptides were added in the following order: IREDIAGVVVF, ATLIDPKRGHVG, YVVIPSTE, REDIT, TDHP, and EEY.

As shown in Figure 3, an increasing umami sensory score was observed in the control samples without peptide addition as fermentation progressed. Assessment of sample differences in repeated-measures umami sensory data: effect sizes and confidence intervals, panelist effects, and model diagnostics (see Supplementary File S3). Sample A on day 0 of wort fermentation scored 2.34. Sample B on day 1 scored 3.19. Sample C on day 3 scored 4.61. Sample D on day 9 reached 6.44. The increase from A to B was 0.85. The increase from B to C was 1.42. The increase from C to D was 1.83. These overall sensory data indicated that umami in lager beer did not form only once at the early stage of processing. It was closely coupled with the fermentation course and showed a clear time dependent accumulation. This trend was consistent with the gradual release of amino acids and small peptides during beer fermentation [75,76]. It was also consistent with the reshaping of acid and ionic systems. In addition, it matched the sustained influence of yeast metabolites on taste interactions.

After 6 representative umami peptides were individually added to 4 process stage beer samples, a distinct stratification was observed. The long chain peptides IREDIAGVVVF and ATLIDPKRGHVG showed negative changes at all stages. Differences were calculated relative to the corresponding control. For IREDIAGVVVF, the values in A, B, C, and D were −0.74, −2.19, −1.51, and −2.44. For ATLIDPKRGHVG, the values were −0.64, −1.19, −2.81, and −2.44. The negative magnitude was more pronounced in the middle and late stages. The medium length peptide YVVIPSTE was also negative throughout. The values in A, B, C, and D were −1.24, −0.39, −1.61, and −2.54. The largest decrease occurred at the mature stage D. In contrast, the short chain peptides showed an overall gain. Noticeable increases were observed for REDIT and TDHP at multiple stages. For REDIT, the values in A, B, C, and D were +0.99, +0.31, +0.09, and +0.86. A positive effect was maintained across nearly the entire process. TDHP showed the strongest gain in A and D. The value was +1.49 in A and +1.36 in D. A decline of −0.41 was observed in C. EEY was positive in A, B, and D with values of +0.49, +0.31, and +0.66. It was −0.21 in C. When the best addition effect at each stage was considered, the highest scores in A and D were obtained with TDHP and reached 3.83 and 7.8. REDIT showed a slight advantage in B and C and reached 3.5 and 4.7. Overall, 10 of 12 additions in the short chain group were positive. The long chain and medium chain groups were negative throughout. This consistency suggested that the contribution of umami peptides to beer umami was not determined by higher docking scores alone. It was more likely defined by an effective window shaped jointly by structural features and the matrix environment.

From a mechanistic perspective, short chain peptides more readily became perceptible contributors to umami. The key reason was that they were closer to the recognition preference of taste receptors and remained accessible in the complex beer matrix. Umami was mainly mediated by the heterodimer formed by T1R1 and T1R3. Ligand selection depended not only on binding strength. It also depended on whether conformational changes could be triggered and downstream signaling could be initiated. Previous studies reported that T1R1 preferred relatively small peptide ligands. Acidic and hydrophilic features supported recognition and stable binding. Structural summaries of umami peptides also indicated that many umami peptides were dipeptides and tripeptides. They often contained negatively charged residues such as Asp or Glu [77,78]. Consistent with this pattern, short chain peptides such as REDIT, TDHP, and EEY were more likely to reach the taste bud surface at a higher effective concentration and with faster diffusion. Receptor occupation could therefore be achieved more efficiently. Sensory perception was expressed as enhanced umami or a clearer umami profile. Meanwhile, beer umami was not an isolated pathway. A kokumi pathway in the oral cavity could enhance mouthfulness, continuity, and overall savoriness. CaSR could be activated by glutathione and multiple γ glutamyl peptides. It enhanced intensity and persistence in umami and salty solutions. The representative peptides in the present study were not necessarily classical γ glutamyl peptides. However, multi-site actions of short chain peptides in the oral cavity could still produce a similar mouthfulness synergy. This effect could be amplified in the mature stage D where the basal umami was higher [79,80].

It was noteworthy that the gain from short chain peptides weakened or even became negative at stage C. Day 3 often corresponded to a window of high fermentation intensity. More suspended yeast and colloidal particles were present in the samples. Carbon dioxide and the acidity system also changed rapidly. Masking and interaction effects of the matrix on taste perception were amplified. Matrix effects in beer have been repeatedly emphasized in taste research. Alcohol content, pH, residual extract, carbon dioxide, iso α acids, and polyphenols could alter perception thresholds and attention allocation for bitterness and mouthfeel. Umami expression could therefore be indirectly changed. In this dynamic matrix, short chain peptides could still show a reduced sensory gain even when umami potential existed. This reduction could occur because the peptides were taken up and utilized by the yeast peptide transport system over a short period. It could also occur because noncovalent binding with charged colloids and polyphenols reduced the free fraction. Binding and precipitation between polyphenols and peptides formed a key molecular basis for oral astringency and beverage haze. Related studies showed that polyphenols could form complexes with peptide chains and precipitate [75,76]. Protein polyphenol complexes are also typical in beer. This mechanism provided a reasonable path to explain the decrease in effective concentration of short chain peptides at mid fermentation. Because of this matrix gating effect, REDIT remained slightly positive at stage C. TDHP and EEY became negative. This pattern suggested differences among short chain peptides in charge distribution, hydrophobicity, and the mode of interaction with the matrix [81,82,83]. These differences determined whether a peptide was expressed or masked at a given time point.

It should be emphasized that higher docking scores for long chain umami peptides did not necessarily lead to a positive effect on beer umami. Strong evidence was provided by the experimental data. The key point was that docking scores mainly reflected binding tendency under static structures. Taste perception was a rapid, competitive, and strongly matrix dependent dynamic process. Higher docking scores of long chain peptides often arose from larger contact areas and more hydrogen bonding or hydrophobic interactions. Such binding could be closer to an occupancy type interaction. Partial agonism or antagonism could also be presented. Effective conformational closure and signal amplification required for T1R1 and T1R3 could therefore fail to occur. In addition, long chain peptides more easily carried continuous hydrophobic residue segments. Studies on bitter peptides commonly indicated a close link between peptide bitterness and enrichment of hydrophobic residues. Increased hydrophobicity at the C terminus markedly elevated bitterness risk [74,81]. Bitterness in the beer matrix could be further amplified by alcohol content and pH. Subjective attention to umami could be suppressed through taste interactions. Moreover, long chain peptides were more likely to form more stable complexes with polyphenols or be adsorbed by the foam protein network. The free fraction decreased. The effective dose reaching the receptor became lower. Therefore, the coexistence of high docking scores and persistent negative sensory outcomes for long chain peptides was not contradictory. A conclusion more consistent with the real beer system was indicated. Umami enhancement was determined not by maximizing binding scores. It was determined by selecting small peptide windows that maintained a free fraction in the beer matrix, showed low off taste risk, and triggered receptor agonism or mouthfulness synergy.

### 3.6. Distribution of Umami Peptide Clusters and Correlation with Umami Sensory Scores in Lager Beer

Based on the analyses above, the umami peptides screened by molecular informatics were supported as scientifically reasonable candidates. Umami peptides identified across the 40 samples were further semi-quantified, and sensory evaluation was conducted. The results are shown in Table 3. Across the 4 brewing nodes, lager beer showed a clear gradient in umami peptide content. Umami sensory scores increased in parallel. An overlap in the content ranges between wort fermentation day 9 and day 0 was still observed. However, sensory scores were overall higher at day 9. This pattern indicated that umami expression was not determined solely by a linear relationship with total content. Peptide composition and matrix synergy likely contributed to amplification of perceived differences. In addition, the late fermentation interval showed a distinct quantitative jump that was consistent with a possible contribution from yeast autolysis. Total umami peptide concentration increased from 196,149.89 μg/L at day 3 to 229,657.79 μg/L at day 9. The increment was 33,507.90 μg/L, which corresponded to a 17.08% increase relative to day 3. This late-stage increment accounted for 32.39% of the net increase from day 0 to day 9. In parallel, the umami sensory score increased by 1.83 from day 3 to day 9, which represented 44.63% of the total sensory gain across the full course. These quantitative features supported that late-stage cell turnover and partial autolysis could have added additional peptide inputs and reshaped the effective composition, beyond earlier accumulation alone. Across the 4 brewing nodes, the Pearson correlation coefficient was r = 0.963 with *p* = 0.037. This result indicated a significant positive correlation between umami peptide accumulation and umami scores at the node scale, with strong linear consistency.

At the mechanistic level, this statistical relationship was explained by gradual accumulation of umami peptides as fermentation progressed. Effective ligand supply to the extracellular VFT recognition cavity of T1R1/T1R3 was thereby increased, which raised the probability of stable receptor activation. Peptides were typically positioned through multipoint weak interactions. Charged groups enabled charge pairing and hydrogen-bond anchoring, whereas hydrophobic side chains promoted pocket complementarity and conformational convergence. As nodes advanced, total abundance increased and sequence combinations more compatible with the pocket were also more likely to emerge. The tendency for VFT closure was therefore enhanced, and downstream signal transduction was triggered more efficiently. The importance of umami peptides in lager beer was not limited to increasing umami intensity. Umami peptides also provided a more persistent and fuller taste framework. Through synergy with other taste-active substances, a more stable umami profile and smoother drinking experience were shaped.

### 3.7. Umami Peptide Clusters Across the Full Lager Beer Brewing Process and Database Construction

From a mechanistic perspective, the system-level umami output was closer to a jointly shaped outcome of multiple ligands rather than an exclusive contribution from a single representative peptide. In addition, members of the glutamate receptor family were reported to potentially contribute to umami or glutamate-related signaling in taste bud cells. Umami therefore appeared to involve parallel receptors with complementary roles at the peripheral level.

This group-encoding logic was highly similar to the olfactory system. In olfaction, a combinatorial coding framework had long been proposed. A single odorant could activate multiple olfactory receptors, and a single receptor could respond to a set of structurally similar or partially compatible ligands. Perception was ultimately determined by the receptor activation pattern. This framework provided stronger explanatory power when transferred to umami peptide research. In beer, peptides originated from multisite and quasi-random cleavage of raw-material proteins, followed by further processing. A single taste direction was therefore often carried by a cluster of peptides with similar sequences, similar physicochemical properties, and similar receptor interaction modes. In other words, the functional unit of umami peptides was more reasonably defined as an umami peptide cluster rather than a single peptide sequence.

Based on this rationale, an “umami peptide cluster” was defined operationally as a group of peptides that were similar in (a) primary sequence length and composition and (b) physicochemical property profiles relevant to umami receptor engagement, rather than as a single peptide identity. Construction of clusters followed an explicit workflow. First, candidate umami peptides were extracted from Supplementary File S2 using the model outputs from UMPred-FRL and TastePeptides-Meta, where each peptide was associated with a predicted umami probability score (default decision threshold 0.5 in UMPred-FRL) and corresponding predicted labels and scores from TastePeptides-Meta. Second, each peptide was encoded into a feature vector combining sequence-derived variables and structure-informed descriptors. Sequence-derived variables included peptide length, molecular weight, net charge proxy based on acidic residue counts, counts or fractions of Asp/Glu as anionic sites, and counts or fractions of hydrophobic residues (e.g., Ile/Val/Leu) reflecting pocket complementarity potential. Structure-informed descriptors were computed from SMILES using RDKit-style 2D descriptors, including MinEStateIndex, SMR_VSA, BCUT2D_MWLOW/BCUT2D_MWHI, VSA_EState1–10, EState_VSA1–11, PEOE_VSA1–14, and MolLogP. These descriptors summarized size, partial-charge distribution, and surface-area partitioning patterns in a standardized, model-ready form. Third, all variables were scaled (z-score) prior to clustering to avoid dominance by any single magnitude range. Clustering was performed on the standardized feature matrix using an unsupervised agglomerative strategy, and the cluster cut point was selected to maximize within-cluster similarity while maintaining separation between clusters based on internal validity indices (e.g., silhouette-style separation). “Cluster nodes” in the subsequent full-process analysis corresponded to these finalized clusters. Each peptide was assigned to exactly one cluster node, and node-level abundance in each sample was calculated by summing the semi-quantified intensities of peptides belonging to that node. Cluster labels were reported using representative centroid-like peptides together with the dominant feature signatures (length bin and key residue pattern) to preserve interpretability. Finally, docking-derived interaction patterns were used as an annotation layer to support mechanistic interpretation of clusters, but docking scores were not used as the primary variable for defining clusters.

On this basis, umami peptides in Supplementary File S2 were incorporated into a full-process umami peptide cluster database for lager beer. Chemical identifiers were provided for umami peptides, including SMILES, MinEStateIndex, SMR_VSA, BCUT2D_MWLOW, BCUT2D_MWHI, VSA_EState1–10, EState_VSA1–11, PEOE_VSA1–14, and MolLogP. Brief sensory descriptions were also provided. These outputs were compiled as Supplementary Files S4 and S5.

## 4. Discussions

### 4.1. Matrix Effects, Ion Suppression, and Detection Bias in Beer Peptidomics

Beer is a chemically dense and dynamically changing matrix across fermentation, and this complexity can influence both what is truly present and what is detectable by LC–MS. In electrospray-based workflows, co-eluting salts, organic acids, residual extract components, iso-α-acids, and polyphenols can contribute to ion suppression and differential response factors, which can bias detection toward peptides that are more abundant, more readily ionizable, or chromatographically favored. This methodological reality is consistent with our sensory-side observation that matrix-driven masking and interaction effects are amplified in mid-fermentation (Day 3) when suspended yeast/colloids, CO_2_, and acidity change rapidly, reshaping perceptual thresholds and attention allocation across taste modalities. Importantly, matrix effects are not only analytical; they also affect the effective free fraction of peptides available for taste perception.

A key source of bias in both measurement and perception arises from noncovalent and colloidal interactions. The manuscript explicitly recognizes that polyphenol–peptide complexation and precipitation can reduce the free fraction of peptides and provides a mechanistic explanation connecting these interactions to oral astringency and beverage haze. In the same logic, adsorption into foam-related protein networks can further decrease the fraction that reaches receptors, providing a realistic pathway by which peptide signals can be underrepresented (analytically) or suppressed (sensory) even when peptides are present. Therefore, throughout the discussion we interpret peptidomic outputs as semi-quantitative and matrix-conditioned, and we emphasize stage-wise comparisons and converging evidence (semi-quant trends + sensory + spiking outcomes) rather than treating LC–MS signal intensity as a direct proxy for sensory potency.

### 4.2. Docking Results vs Sensory Intensity: Binding Affinity ≠ Perceived Umami

A central conclusion supported by the validation data is that docking-derived binding propensity cannot be treated as a proxy for perceived umami, particularly in beer. This is demonstrated directly by the spiking experiment: across stages, 10 of 12 additions in the short-chain group were positive, whereas the long- and medium-chain groups were negative throughout, indicating that stronger or more extensive receptor contact predicted by docking does not guarantee positive sensory outcomes. The same dataset supports a more realistic “effective window” concept in which perceptible umami enhancement emerges only when structural features and the beer matrix jointly allow sufficient free fraction, low off-taste risk, and receptor-productive signaling.

Docking scores primarily reflect a static binding tendency under a fixed receptor conformation, while taste perception is rapid, competitive, and strongly matrix-dependent. The manuscript now explicitly notes that larger contact areas in long peptides can yield higher docking scores yet correspond to occupancy-like binding that may fail to induce the conformational closure and downstream signaling required for T1R1/T1R3 activation; partial agonism or antagonism is also plausible. In addition, long peptides more readily contain extended hydrophobic segments that can elevate bitterness risk, and bitterness can be further amplified by alcohol content and pH, suppressing perceived umami through taste interactions. Together, these points justify a cautious interpretation: docking is valuable for hypothesis generation and interaction mapping, but sensory relevance must be established experimentally, as performed here.

### 4.3. Competitive Binding and Co-Ligand Context: Glutamate, Nucleotides, and Ethanol Effects

The receptor-level framework also suggests that peptide effects should be interpreted in the context of co-existing ligands in beer. T1R1/T1R3 recognition is not determined by a single residue cluster; multiple sites within the VFT and near the interface can jointly contribute to ligand anchoring and conformational stabilization. The manuscript further emphasizes that, in addition to free amino acids, umami peptides derived from food protein hydrolysis are proposed to interact with T1R1/T1R3, providing a plausible receptor basis for peptide binding modes. Under real brewing conditions, free amino acids (including glutamate released by proteolysis and turnover) and umami-enhancing nucleotides are expected to be present to varying extents across stages; thus, competitive occupancy, allosteric modulation, and mixture-dependent receptor coding are highly relevant to perceived output.

From the sensory side, stage dependence further argues for mixture and matrix competition. Day 3 represents a window where matrix masking is amplified by suspended yeast/colloids and rapidly shifting CO_2_/acidity, such that even peptides with umami potential can show reduced gains. In addition, alcohol content and pH can amplify bitterness and suppress attention to umami via taste–taste interactions, reinforcing that receptor binding alone is insufficient to predict perceived intensity. Therefore, we frame umami as an emergent property of multi-ligand competition + matrix gating + receptor-productive signaling, rather than a monotonic function of any single ligand class.

### 4.4. Linkage to Beer Flavor Literature: Peptides/Polypeptides, Body Fullness, and Smoothness

To better connect umami peptides to beer quality, it is important to embed umami within the broader sensory constructs of body fullness, continuity, and smoothness. The manuscript explicitly recognizes that beer umami is not an isolated pathway and discusses a mouthfulness-synergy route consistent with kokumi-like enhancement, where CaSR activation by glutathione and γ-glutamyl peptides can increase persistence and savoriness; while the representative peptides here are not necessarily classical γ-glutamyl peptides, multi-site actions of short peptides may still produce analogous mouthfulness synergy, particularly when basal umami is higher. This provides a beer-relevant framing in which umami peptides contribute not only to “umami intensity” but also to texture-linked savoriness.

At the same time, beer mouthfeel is strongly shaped by colloidal and macromolecular interactions, which can modulate smoothness and fullness while also gating taste availability. Polyphenol–peptide binding and precipitation are highlighted as a molecular basis for oral astringency and haze, and protein–polyphenol complexes are described as typical in beer. These interactions provide a coherent explanation for stage-specific sensory outcomes (e.g., weakening or reversal of peptide gains at Day 3) and reinforce why perceptual umami must be interpreted as matrix-conditioned rather than solely receptor-ligand determined. Finally, the node-level finding that umami peptide content and sensory scores rise in parallel, yet still show partial overlap in content ranges between Day 9 and Day 0, indicates that matrix synergy and composition-dependent amplification remain non-negligible contributors beyond peptide abundance alone. This strengthens the linkage between peptide chemistry and beer-quality perception, while maintaining an appropriately cautious mechanistic interpretation.

## 5. Limitations

Semi-quantification was performed using a single synthetic peptide standard (PVPL) on an RPLC-Q-TOF-MS platform. This strategy provided a practical and consistent scaling reference across batches. However, peptide MS responses are sequence dependent and can vary substantially with physicochemical properties, including peptide length, charge state, and hydrophobicity, which influence ionization efficiency and signal response in ESI. Therefore, PVPL-based semi-quantification might not fully capture peptide-to-peptide response-factor variability across peptides with distinct compositions and lengths. The quantitative outputs were therefore interpreted as semi-quantitative trends rather than absolute concentrations. Importantly, the analytical workflow used uniform sample preparation and consistent RPLC-Q-TOF-MS settings across all samples, which supported reliable relative comparisons within the study design. Thus, despite this limitation, the approach remained suitable for identifying stage-dependent abundance trends and prioritizing candidates for downstream validation. Future work could further reduce response-factor uncertainty by including multiple calibrant peptides spanning representative sequence properties or by employing stable isotope-labeled internal standards for peptide-specific correction.

The contribution of yeast-derived peptides remained underexplored relative to cereal sources. Beer proteomic studies have shown that the beer proteome contains components originating from both barley and yeast, and that the proteome changes dynamically across processing steps. During fermentation and downstream maturation, yeast physiology can reshape the peptide pool through uptake and metabolism of wort peptides, secretion of proteins, and autolysis-associated release of intracellular compounds, including peptides and amino acids. As a result, peptides detected in finished beer may reflect mixed origins and process-driven remodeling rather than cereal inputs alone. Within the current design, peptide origin was not systematically partitioned between cereal and yeast sources, and yeast strain-dependent effects were not explicitly evaluated. This constraint did not invalidate the main conclusions regarding process-dependent trends and candidate prioritization, but it limited mechanistic attribution of specific peptides to yeast versus cereal origins. Future work should strengthen source assignment by integrating strain-specific proteogenomic databases, time-resolved profiling from wort to beer, and targeted confirmation of yeast-specific peptide markers, as well as experimental strategies that facilitate origin tracing, such as isotopic labeling or controlled fermentations with defined yeast contributions.

The predictive framework integrated peptidomics, machine-learning taste predictors, clustering, and docking annotations, but several constraints remained (Supplementary File S3). First, sequence-based umami predictors such as UMPred-FRL and TastePeptides-Meta were trained on curated datasets with heterogeneous experimental conditions, and their probability outputs (e.g., a 0.5 decision threshold in UMPred-FRL) represented classification likelihood rather than sensory intensity in a beer matrix. Second, docking provided a static, model-dependent snapshot of binding poses and scoring, and score magnitude could not be treated as a direct proxy for receptor activation or perceived umami because receptor dynamics, competitive ligands, and matrix-dependent bioavailability were not captured. Third, the clustering scheme depended on selected variables and scaling choices, and although it improved interpretability at the node level, cluster boundaries could shift if alternative descriptor sets or distance metrics were applied. Therefore, the final model was interpreted as a robust prioritization and mechanistic organization tool within this brewing system, while causal attribution of umami to any single peptide or cluster still required targeted sensory confirmation and quantitative validation.

## 6. Conclusions

This study used the full brewing process as the primary framework and integrated peptidomics identification, molecular informatics screening, receptor-level docking, and sensory validation into a single, cross-level evidence chain. In this way, the work supports a shift from purely outcome-based description of umami in lager beer toward mechanistic hypothesis development and database-based representation. Across the full process, 25,592 peptides were identified and 7255 potential umami peptides were screened. Collectively, these findings suggest that fermentation progression is not merely a unidirectional degradation endpoint; rather, the peptide pool appears to remain dynamic, with continuous generation and redistribution across nodes. A total of 145 peptides were further selected for molecular docking, and six representative umami peptides were subsequently chosen for receptor-level validation and structural interpretation.

At the receptor-recognition level, docking results suggested that several peptides could adopt geometries compatible with the recognition cavity through hydrogen bonding and electrostatic interactions at residues such as Lys228, Arg240, Glu206, Asp210, Asn141, and Gln138, with additional stabilization potentially supported by a hydrophobic boundary including Val135, Ile137, Leu165, Tyr166, Trp78, and His79. These interaction patterns are consistent with the mechanistic plausibility that certain ligand poses may facilitate VFT closure and downstream signaling, but they do not establish receptor activation directly. Energy decomposition analyses further indicated that nonpolar terms were major contributors to predicted binding gains, whereas net electrostatic terms often manifested as energetic costs across multiple peptides. Importantly, docking scores were used primarily for relative pose ranking and hypothesis generation rather than as a proxy for perceived umami intensity; binding tendency alone does not guarantee receptor efficacy because productive activation depends on whether ligand engagement promotes VFT closure and signaling.

At the node scale, sensory results showed a strong positive correlation (r = 0.963, *p* = 0.037), which is consistent with an association between umami-peptide accumulation and elevated umami perception across process nodes. However, overlap in content ranges across nodes could still occur while sensory scores continued to rise, suggesting that peptide composition and matrix-level interactions may contribute to perceptual amplification beyond total peptide content alone. Accordingly, rather than attributing umami to a few representative sequences, a more plausible interpretation is that receptor recognition may involve broad-spectrum, multisite responses, and that system-level umami is jointly carried by peptide clusters sharing structural and physicochemical features. In addition to umami intensity, peptides and polypeptides may also contribute to body, smoothness, and mouthfeel persistence, potentially conditioning how umami is expressed within the beer matrix rather than acting as an isolated taste channel.

Accordingly, the umami peptide cluster database is best viewed as an evidence-organizing and hypothesis-indexing framework for interpreting node-to-node differences within the studied brewing system, rather than as a universal process-optimization solution. Further calibration via absolute quantification, orthogonal receptor functional assays, and controlled reconstitution or spiking designs will be necessary before cluster-level outputs can be translated into robust and transferable control rules for brewing.

## Figures and Tables

**Table 1:** Docking binding energies between 145 umami peptides and the receptor T1R1/T1R3.

Number	Peptide	Docking Score	Glide Ecoul	Glide Emodel	Glide Energy	Glide Evdw
1	AAEVIE	−7.137	−22.642	−99.999	−63.578	−40.936
2	AAEVLELAGNAAKDNKK	−9.750	−25.095	−143.533	−117.206	−92.111
3	ADA	−5.164	−13.834	−54.561	−38.664	−24.830
4	ADV	−6.696	−13.480	−55.502	−37.597	−24.116
5	AE	−5.814	−21.309	−48.935	−36.280	−14.971
6	AER	−6.441	−22.681	−75.253	−48.681	−26.000
7	AES	−7.290	−18.546	−69.127	−44.300	−25.754
8	AGDM	−6.624	−18.327	−72.288	−48.996	−30.669
9	ARE	−7.086	−19.919	−70.491	−42.190	−22.271
10	ARGVE	−5.947	−36.287	−96.658	−61.684	−25.398
11	ATATQAP	−7.581	−22.030	−109.554	−74.253	−52.223
12	ATEPY	−6.959	−20.566	−95.375	−63.719	−43.153
13	ATLIDPKRGHVG	−10.260	−32.393	−183.914	−105.181	−72.788
14	ATTSI	−6.699	−21.079	−81.635	−57.563	−36.485
15	ATTSIA	−8.026	−23.567	−104.869	−75.755	−52.188
16	AVD	−5.378	−16.191	−58.720	−43.676	−27.485
17	AVSTST	−8.199	−20.078	−102.698	−69.582	−49.505
18	DKRQ	−6.990	−28.114	−99.981	−65.913	−37.799
19	DMVH	−5.719	−19.331	−85.437	−63.413	−44.083
20	DQ	−3.796	−16.671	−45.451	−36.512	−19.841
21	DYDYGSNTCAAGKVC	−12.568	−31.511	−180.057	−127.553	−96.042
22	EA	−6.815	−20.975	−54.720	−36.756	−15.781
23	EAGM	−5.730	−18.267	−68.397	−53.026	−34.759
24	EARQ	−6.722	−24.508	−87.294	−58.107	−33.599
25	EATT	−6.799	−19.583	−69.140	−49.936	−30.353
26	EAV	−6.103	−19.066	−59.557	−43.043	−23.977
27	EDKAG	−6.045	−34.290	−94.726	−64.597	−30.307
28	EDRY	−6.552	−21.485	−93.845	−58.360	−36.875
29	EEH	−7.252	−30.103	−91.655	−61.870	−31.768
30	EEY	−8.194	−20.756	−78.486	−53.394	−32.638
31	EGAY	−6.554	−25.172	−77.289	−57.272	−32.100
32	EGM	−6.260	−21.278	−67.076	−51.959	−30.681
33	EGMP	−6.156	−11.911	−72.779	−56.770	−44.859
34	EGTT	−6.207	−21.693	−74.576	−57.379	−35.685
35	EGVVF	−4.844	−12.354	−76.888	−57.054	−44.700
36	EHE	−7.749	−21.940	−76.627	−49.253	−27.313
37	EK	−3.275	−21.570	−48.041	−38.670	−17.100
38	EKM	−5.694	−16.258	−70.128	−47.939	−31.681
39	EKV	−5.750	−18.397	−65.722	−45.709	−27.311
40	EM	−4.502	−14.605	−49.025	−36.916	−22.312
41	EMQ	−7.130	−27.333	−77.702	−57.061	−29.729
42	EMVT	−6.989	−21.623	−80.787	−62.031	−40.408
43	EN	−6.107	−24.514	−51.998	−38.077	−13.563
44	EPGA	−6.111	−19.513	−68.742	−47.891	−28.378
45	EPGM	−4.911	−13.175	−61.422	−47.404	−34.229
46	EPRW	−7.249	−22.554	−104.975	−63.779	−41.225
47	EPSPV	−4.856	−8.736	−65.325	−51.709	−42.973
48	ESV	−5.995	−19.081	−62.593	−43.406	−24.325
49	ESY	−7.301	−20.100	−71.643	−48.955	−28.854
50	ETV	−5.884	−15.109	−59.757	−42.487	−27.378
51	ETY	−6.458	−15.818	−66.692	−50.478	−34.661
52	EVDPAP	−7.639	−17.529	−95.877	−65.996	−48.467
53	EVG	−5.043	−15.868	−52.048	−36.297	−20.428
54	EVHE	−6.521	−17.290	−82.206	−57.636	−40.346
55	EVK	−6.811	−17.403	−67.043	−44.445	−27.042
56	EVKST	−6.677	−21.940	−90.152	−60.175	−38.235
57	EVV	−5.857	−22.182	−59.406	−45.776	−23.594
58	FVEVNEEGTEAGAATVA	−7.238	−15.929	−119.739	−92.511	−76.583
59	GDCSG	−6.786	−22.201	−85.066	−59.170	−36.969
60	GHVPE	−7.233	−20.887	−99.690	−64.373	−43.487
61	HASPR	−7.134	−21.589	−99.644	−64.017	−42.427
62	HDT	−8.069	−25.302	−78.827	−54.950	−29.648
63	HSME	−7.144	−21.762	−93.380	−70.362	−48.600
64	IREDIAGVVVF	−10.348	−33.774	−168.065	−100.074	−66.300
65	KATHC	−8.313	−27.680	−123.280	−67.577	−39.897
66	KCGAN	−7.268	−25.532	−97.654	−64.458	−38.926
67	KDTSG	−8.282	−26.949	−100.510	−66.699	−39.750
68	KDTSH	−8.564	−23.701	−121.779	−77.339	−53.638
69	KEGRE	−7.220	−25.490	−102.591	−63.441	−37.951
70	KRAE	−5.506	−37.104	−101.314	−61.790	−24.686
71	KTEGA	−8.210	−29.028	−95.507	−66.444	−37.416
72	LALDSTGVFKEL	−9.467	−19.933	−146.930	−86.504	−66.571
73	LAQMEAIRTL	−9.560	−29.314	−141.617	−90.907	−61.594
74	LDRKE	−8.120	−24.907	−116.512	−67.721	−42.814
75	LNFTGWVDVK	−7.690	−34.868	−136.627	−97.281	−62.413
76	MDM	−6.193	−21.219	−71.369	−51.221	−30.002
77	MDR	−6.615	−18.220	−73.255	−46.102	−27.882
78	MEIGPLDTELKPR	−8.644	−22.084	−124.407	−83.008	−60.924
79	MGE	−6.846	−21.893	−65.022	−49.464	−27.571
80	MKE	−5.881	−16.321	−72.120	−51.888	−35.567
81	MRE	−7.007	−20.054	−83.317	−48.986	−28.932
82	NIFLDSR	−8.940	−27.594	−134.528	−83.635	−56.041
83	NSGD	−6.508	−21.082	−82.648	−52.869	−31.787
84	NVNDVLAPAFVK	−9.448	−18.778	−141.216	−93.966	−75.189
85	PDMGG	−6.687	−19.682	−81.513	−58.148	−38.466
86	PHDR	−7.031	−20.591	−91.249	−56.630	−36.039
87	PHDT	−5.941	−17.700	−77.574	−52.289	−34.589
88	PHRE	−8.445	−25.521	−103.287	−57.501	−31.980
89	PSTV	−5.923	−20.825	−67.426	−46.087	−25.262
90	PSVP	−5.194	−9.793	−58.968	−44.392	−34.599
91	PTE	−6.667	−17.441	−58.647	−45.230	−27.789
92	QCQ	−6.929	−18.921	−71.301	−52.549	−33.628
93	QLAQMEAIRTLVL	−11.805	−31.549	−183.969	−112.209	−80.661
94	QPQQQVPVEVM	−9.882	−31.693	−156.254	−100.514	−68.822
95	QQMNTCAFLQQCSRT	−9.842	−20.219	−172.146	−114.876	−94.656
96	QSKTAA	−8.569	−21.893	−117.216	−73.016	−51.124
97	RAPN	−5.679	−20.414	−80.136	−51.606	−31.192
98	RDM	−5.462	−21.523	−70.894	−48.738	−27.215
99	REAPPSG	−7.738	−21.924	−122.357	−69.371	−47.447
100	REDIT	−8.385	−32.199	−116.532	−69.401	−37.202
101	REGMQ	−8.477	−30.709	−122.673	−71.915	−41.207
102	RGDS	−7.647	−28.092	−89.018	−55.539	−27.446
103	RHTAP	−7.376	−28.455	−105.845	−62.825	−34.370
104	RMD	−5.368	−17.167	−70.902	−48.772	−31.605
105	RSTGPA	−7.037	−19.554	−104.849	−58.460	−38.905
106	SGRC	−7.986	−25.968	−88.446	−51.479	−25.511
107	SSF	−6.404	−21.063	−71.206	−51.853	−30.790
108	SSME	−7.652	−23.137	−76.509	−56.996	−33.859
109	STTKM	−6.892	−19.238	−92.362	−63.215	−43.977
110	SVE	−5.154	−16.609	−55.418	−40.959	−24.350
111	TAAAGAGAGT	−10.066	−29.510	−140.096	−89.341	−59.831
112	TAE	−5.611	−14.006	−61.029	−44.410	−30.404
113	TAMD	−6.739	−18.378	−79.836	−54.904	−36.526
114	TATGG	−6.674	−24.190	−80.073	−54.824	−30.634
115	TDHP	−9.113	−24.355	−91.571	−66.289	−41.934
116	TDRG	−7.474	−25.797	−88.830	−55.280	−29.483
117	TE	−3.336	−18.740	−44.432	−37.050	−18.309
118	TGVV	−5.521	−13.646	−67.992	−48.441	−34.795
119	THDM	−7.671	−27.313	−96.983	−65.324	−38.012
120	TMKS	−7.028	−16.167	−79.780	−52.468	−36.301
121	TPE	−4.141	−16.506	−53.030	−44.342	−27.835
122	TSKN	−6.482	−23.276	−83.596	−58.737	−35.461
123	TSTR	−7.148	−27.088	−84.011	−51.036	−23.949
124	TTMP	−6.658	−19.588	−71.027	−60.798	−41.210
125	TVEVV	−5.546	−21.101	−81.582	−57.378	−36.278
126	TVFGT	−6.929	−21.372	−93.709	−60.902	−39.530
127	TVIATGEYKEPITQ	−9.008	−20.298	−136.170	−98.705	−78.407
128	TVKTS	−7.754	−17.683	−99.745	−65.650	−47.967
129	TVSF	−6.735	−13.981	−70.943	−52.510	−38.529
130	TVSHD	−7.622	−25.355	−100.326	−62.352	−36.997
131	TVTVP	−6.130	−26.878	−81.151	−59.778	−32.900
132	VAVND	−5.913	−15.094	−83.813	−59.382	−44.288
133	VEVPGGLT	−7.748	−16.342	−105.193	−71.098	−54.756
134	VPAMAT	−8.084	−20.910	−94.676	−67.034	−46.124
135	VSAE	−5.518	−14.582	−60.204	−49.401	−34.819
136	VSPAAT	−8.225	−21.523	−96.424	−68.145	−46.621
137	VSPHQGQQTTVSPHQGQQTT	−9.369	−26.736	−159.672	−117.920	−91.183
138	VTGTY	−6.729	−23.217	−92.520	−65.606	−42.389
139	VTGV	−5.820	−19.319	−65.832	−50.120	−30.801
140	VVIPSTE	−8.053	−19.319	−118.921	−82.753	−63.434
141	YATT	−7.977	−19.863	−80.810	−60.246	−40.383
142	YST	−4.830	−15.544	−57.155	−42.524	−26.980
143	YTAT	−5.999	−14.715	−79.823	−58.757	−44.042
144	YTS	−5.040	−17.259	−62.194	−51.479	−34.219
145	YVVIPSTE	−9.483	−14.735	−131.433	−85.383	−70.648

Note: Docking Score was the primary metric for ligand ranking and screening. Lower values indicated better performance. Units were kcal/mol (same below). Glide Ecoul was the Coulomb (electrostatic) interaction energy between the receptor and ligand. Glide Emodel was used for pose selection and stability comparison within the same ligand and was mainly used to choose among different poses of a given ligand. Glide Evdw was the van der Waals interaction energy between the receptor and ligand derived from the vdW term in the force field.

**Table 2:** MM-GBSA binding energies between six umami peptides and their receptor.

Peptide	MM-GBSA dG Bind (NS)	MM-GBSA dG Bind (NS) Coulomb	MM-GBSA dG Bind (NS) Hbond	MM-GBSA dG Bind (NS) Lipo	MM-GBSA dG Bind (NS) Packing	MM-GBSA dG Bind (NS) Solv GB	MM-GBSA dG Bind (NS) vdW
IREDIAGVVVF	−92.74	54.12	−12.55	−19.40	0.00	−9.57	−105.34
ATLIDPKRGHVG	−67.86	33.35	−9.65	−18.98	−0.95	37.17	−108.80
YVVIPSTE	−41.11	409.43	−10.57	−19.28	−1.11	−259.56	−160.02
REDIT	−60.17	96.33	−6.12	−9.77	−1.90	−81.98	−56.74
TDHP	−35.37	141.77	−10.67	−9.70	0.00	−92.46	−64.31
EEY	−33.18	223.33	−6.87	−12.21	−1.58	−176.77	−59.08

**Notes:** All values were reported in kcal/mol. MM-GBSA dG Bind (NS) was the strain-free binding energy. Energies of the receptor and ligand were calculated after separation from the optimized complex and re-optimization. Conformational strain induced by binding was not included. This term equaled the strain-included binding energy minus the conformational strain energies of the receptor and ligand.; MM-GBSA dG Bind (NS) Coulomb was the electrostatic energy contribution within the strain-free binding energy. It represented the change in Coulomb interactions between the ligand and the protein.; MM-GBSA dG Bind (NS) Hbond was the hydrogen-bond energy contribution within the strain-free binding energy. It reflected energy changes associated with hydrogen bonds formed or disrupted upon binding.; MM-GBSA dG Bind (NS) Lipo was the hydrophobic contribution within the strain-free binding energy. It corresponded to the energy difference associated with changes in hydrophobic surface area during binding.; MM-GBSA dG Bind (NS) Packing was the π–π stacking correction contribution within the strain-free binding energy. It accounted for the effect of specific stacking interactions between π systems such as aromatic rings.; MM-GBSA dG Bind (NS) Solv GB was the solvation contribution within the strain-free binding energy. It was the dielectric solvation energy difference between the complex and the separated states calculated using the generalized Born model.; MM-GBSA dG Bind (NS) vdW was the van der Waals energy contribution within the strain-free binding energy. It described changes in van der Waals attractive potential between the ligand and the protein.In terms of total binding energy ranking, dG Bind (NS) of IREDIAGVVVF was −92.74 and showed the strongest predicted affinity. It was followed by ATLIDPKRGHVG at −67.86, REDIT at −60.17, YVVIPSTE at −41.11, TDHP at −35.37, and EEY at −33.18. It should be noted that peptides differed markedly in length and polarity. Ranking was not determined by a single force term. It was determined by synergy and cancellation among multiple components within the same pocket.

**Table 3:** Umami peptide contents across the full lager beer brewing process and the corresponding umami sensory evaluation scores.

Sample Number	Umami Peptide Concentration (μg/L)	Sample Umami Sensory Evaluation Score
1-1	9908.73	2.70
1-2	11,961.80	2.10
1-3	6670.54	2.00
1-4	6848.00	3.00
1-5	8951.04	2.00
1-6	13,292.08	2.30
1-7	9702.44	2.70
1-8	19,202.58	2.00
1-9	22,177.14	2.30
1-10	17,476.80	2.30
1	126,191.14	2.34
2-1	17,981.13	3.00
2-2	10,353.23	3.10
2-3	22,546.02	3.40
2-4	13,718.13	3.90
2-5	15,814.88	3.50
2-6	27,625.07	3.40
2-7	17,864.60	2.90
2-8	13,438.38	3.00
2-9	23,185.78	2.70
2-10	10,624.64	3.00
2	173,151.87	3.19
3-1	22,447.46	4.50
3-2	19,718.05	5.10
3-3	21,527.88	4.30
3-4	14,661.36	4.70
3-5	19,900.57	4.60
3-6	20,912.48	5.00
3-7	19,414.01	4.40
3-8	21,362.25	4.70
3-9	19,694.56	4.80
3-10	16,511.27	4.00
3	196,149.89	4.61
4-1	27,370.00	7.10
4-2	28,599.88	6.80
4-3	23,430.82	5.90
4-4	24,429.97	6.50
4-5	22,408.02	6.10
4-6	28,115.87	6.20
4-7	23,880.68	6.40
4-8	20,884.51	7.00
4-9	16,080.62	6.40
4-10	14,457.41	6.00
4	229,657.79	6.44

## Data Availability

Data will be made available on request. The data presented in this study are available on request from the corresponding author due to space limitations and to maintain the conciseness and readability of the manuscript.

## Supplementary Materials

The following supporting information can be downloaded at: https://www.mdpi.com/article/10.3390/foods15040641/s1, Supplementary File S1: Polypeptide FASTA Format Converter; Supplementary File S2: Qualitative, Semi-Quantitative Indices and Umami Taste Prediction Results of Polypeptides in Lager Beer Samples from Wort Fermentation on Days 0, 1, 3, and 9; Supplementary File S3: Assessment of sample differences in repeated-measures umami sensory data effect sizes and confidence intervals, panelist effects, and model diagnostics; Supplementary File S4: Database of Umami Taste Peptide Clusters in the Complete Lager Beer Brewing Process; Supplementary File S5: Database of Umami Taste Peptide Clusters in the Complete Lager Beer Brewing Process-PDF.

## Abbreviations

The following abbreviations are used in this manuscript:

**Abbreviation**	**Full Term/Meaning**
ABV	Alcohol by volume
ACN	Acetonitrile
ALC	Average local confidence (PEAKS de novo sequencing metric)
AMP	Adenosine 5′-monophosphate
ANOVA	Analysis of variance
BCUT2D_MWHI	RDKit BCUT2D descriptor (MW-high)
BCUT2D_MWLOW	RDKit BCUT2D descriptor (MW-low)
CaSR	Calcium-sensing receptor
EIC	Extracted ion chromatogram
ESI	Electrospray ionization
EState_VSA1-11	RDKit EState_VSA descriptors (bins 1–11)
FA	Formic acid
FASTA	FASTA sequence format
GMP	Guanosine 5′-monophosphate
GPCR	G protein-coupled receptor
GS1	Ion source gas 1 (SCIEX source parameter)
GS2	Ion source gas 2 (SCIEX source parameter)
Glide	Glide (Schrödinger) docking program
IBU	International Bitterness Units
IDA	Information dependent acquisition (SCIEX acquisition mode)
IMP	Inosine 5′-monophosphate
ISO	International Organization for Standardization
LC-MS/MS	Liquid chromatography–tandem mass spectrometry
LigPrep	LigPrep (Schrödinger) ligand preparation module
MM-GBSA	Molecular mechanics generalized Born surface area
MS1	MS1 (precursor/full-scan mass spectrum)
MS2	MS2 (tandem MS / fragment mass spectrum)
MSG	Monosodium glutamate
Maestro	Maestro (Schrödinger) interface
MinEStateIndex	RDKit descriptor: MinEStateIndex
MolLogP	RDKit descriptor: MolLogP (logP estimate)
PEAKS	PEAKS Studio software
PEOE_VSA1-14	RDKit PEOE_VSA descriptors (bins 1–14)
RCSB	Research Collaboratory for Structural Bioinformatics (RCSB Protein Data Bank portal)
RDKit	RDKit cheminformatics toolkit
RMSD	Root-mean-square deviation
SAVES	Structure Analysis and Verification Server (SAVES)
SD	Standard deviation
SMILES	Simplified Molecular Input Line Entry System
SMR_VSA	RDKit SMR_VSA descriptors
SP	Standard Precision (Glide docking mode)
T1R1/T1R3	T1R1/T1R3 umami receptor heterodimer
TBtools	TBtools software
TF	Analyst TF (SCIEX software module used for data processing)
TastePeptides-Meta	TastePeptides-Meta (taste-peptide database & predictor)
UHPLC	Ultra-high-performance liquid chromatography
UMPred-FRL	UMPred-FRL (umami peptide predictor)
UPLC	Ultra-performance liquid chromatography
USA	United States of America
USD	US dollar
UniProt	UniProt (protein sequence database)
UniProtKB	UniProt Knowledgebase
VFT	Venus flytrap (VFT) domain (extracellular ligand-binding domain of class C GPCRs)
VSA_EState1-10	RDKit VSA_EState descriptors (bins 1–10)
XP	Extra Precision (Glide docking mode)
cells/mL	Cells per milliliter
dG	ΔG (Gibbs free energy change)
eV	Electronvolt
g/L	Grams per liter
mGluR1	Metabotropic glutamate receptor 1
vdW	van der Waals
