# Supplementary material for: Molecular Informatics, Chemometrics, and Sensory Omics for Constructing an Umami Peptide Cluster Library Across the Entire Lager Beer Brewing Process
Source: Foods. 2026 Feb 10;15(4):641. doi: 10.3390/foods15040641 (PMC12939766; doi:10.3390/foods15040641)
Supplement: Supplementary file 1 [file foods-15-00641-s001.zip › Supplementary File S1 Polypeptide FASTA Format Converter.html]

多肽 FASTA 格式转换器


# 多肽 FASTA 格式转换器

设计人：武亚帅    指导教师：赵东瑞


输入多肽序列（每行一条，允许前缀编号）：


转换为 FASTA
下载 FASTA 文件

### 转换结果：

下载 FASTA
