# Supplementary material for: Molecular Informatics, Chemometrics, and Sensory Omics for Constructing an Umami Peptide Cluster Library Across the Entire Lager Beer Brewing Process
Source: Foods. 2026 Feb 10;15(4):641. doi: 10.3390/foods15040641 (PMC12939766; doi:10.3390/foods15040641)
Supplement: Supplementary file 1 [file foods-15-00641-s001.zip › Supplementary File S3 Assessment of sample differences in repeated-measures umami sensory....pdf]

**Assessment of sample differences in repeated-measures umami sensory data: effect sizes and confidence intervals, panelist effects, and model diagnostics**

For the fermentation 0 d A-stage sensory scores from six samples (A-1 to A-6), each sample was evaluated once by 10 panelists (n=60). A linear mixed-effects model was fitted to account for the repeated-evaluation structure. Sample was specified as a fixed effect, and panelist was specified as a random intercept to control baseline scoring differences. A significant overall sample effect was observed (Wald $\chi^2$ (5) =14.94, p=0.0106). The estimated marginal means (95% CI) were 1.60 for A-1 (0.84–2.36). They were 1.70 for A-2 (0.94–2.46), 1.10 for A-3 (0.34–1.86), 2.60 for A-4 (1.84–3.36), 2.50 for A-5 (1.74–3.26), and 2.60 for A-6 (1.84–3.36). The highest scores were obtained for A-4 and A-6, whereas the lowest score was obtained for A-3. The largest contrast was observed for A-4 versus A-3, with an increase of 1.50 points (95% CI 0.48–2.52). After standardization by the model residual SD of 1.17, the effect size was d=1.29, indicating substantial practical relevance. The panelist random-effect variance was 0.154, and the residual variance was 1.358, corresponding to ICC=0.10. The comparison between models with and without the panelist random intercept was not significant (likelihood ratio test $\chi^2$ (1) =1.26, p=0.263), indicating limited systematic panelist bias in this dataset. For model explanatory power, the marginal $R^2$ for fixed effects was approximately 0.19, and the conditional $R^2$ after including the panelist random effect was approximately 0.27. Residual diagnostics indicated good homoscedasticity (Levene test p=0.536). No outliers with absolute standardized residuals greater than 3 were detected. A mild deviation from residual normality was detected under strict testing (Shapiro–Wilk p=0.028). Given the bounded discrete scale, inference was primarily reported using effect sizes and their 95% CI.

For the fermentation 1 d B-stage umami scores from samples B-1 to B-6, each sample was evaluated once by 10 panelists (n=60). A linear mixed-effects model was fitted for the repeated-measures structure. Sample was treated as a fixed effect, and panelist was treated as a random intercept. A significant overall sample effect was observed (Wald $\chi^2$ (5) =25.37, $p=1.18\times10^{-4}$). The estimated marginal means (95% CI) were 1.00 for B-1 (0.14–1.86), 2.00 for B-2 (1.14–2.86), 2.80 for B-3 (1.94–3.66), 3.50 for B-4 (2.64–4.36), 3.20 for B-5 (2.34–4.06), and 3.50 for B-6 (2.64–4.36). An overall increasing trend was observed from B-1 to B-4 and B-6. The largest contrast between the highest and lowest samples was observed for B-4 versus B-1, and it also applied to B-6 versus B-1. The mean difference was $\Delta$=2.50 points (95% CI 1.28–3.72). After standardization

by the model residual SD of 1.39, the effect size was d=1.80, indicating substantial practical relevance. The estimated panelist random-effect variance approached 0 (ICC<0.001). Model fit was not improved by including the panelist random intercept (likelihood ratio test $\chi^2$ (1) $\approx$0.00, p$\approx$1.00), indicating negligible systematic panelist bias at this stage. The marginal $R^2$ was approximately 0.30, and the conditional $R^2$ was approximately 0.30. Residual diagnostics supported the model assumptions. Homoscedasticity was satisfactory across groups (Levene test p=0.981). Residual normality showed no significant deviation (Shapiro–Wilk p=0.167). No outliers with absolute standardized residuals greater than 3 were detected.

For the fermentation 3 d C-stage umami scores from samples C-1 to C-6, each sample was evaluated once by 10 panelists (n=60). A linear mixed-effects model was fitted to capture the repeated-measures structure. Sample was treated as a fixed effect, and panelist was treated as a random intercept to control baseline scoring differences. A significant overall sample effect was observed (Wald $\chi^2$ (5) =37.47, p=$4.81\times10^{-7}$). The estimated marginal means (95% CI) were 3.10 for C-1 (2.31–3.89), 1.80 for C-2 (1.01–2.59), 3.00 for C-3 (2.21–3.79), 4.70 for C-4 (3.91–5.49), 4.20 for C-5 (3.41–4.99), and 4.40 for C-6 (3.61–5.19). Clear differentiation in umami intensity was observed across samples at fermentation 3 d. The highest score was obtained for C-4, whereas the lowest score was obtained for C-2. The largest contrast was observed for C-4 versus C-2, with a mean difference of $\Delta$=2.90 points (95% CI 1.79–4.01). After standardization by the model residual SD of 1.27, the effect size was d=2.29, indicating strong practical relevance in addition to statistical significance. The estimated panelist random-effect variance approached 0 (ICC<0.001). Model fit was not improved by including the panelist random intercept (likelihood ratio test $\chi^2$ (1) $\approx$0.00, p$\approx$1.00), indicating negligible systematic panelist bias at this stage. Based on common definitions of marginal and conditional $R^2$, the marginal $R^2$ was approximately 0.39 and the conditional $R^2$ was approximately 0.39, indicating that overall variance was primarily attributable to sample differences. Residual diagnostics supported the main assumptions. Homoscedasticity was satisfactory across groups (Levene test p=0.855). Residual normality showed no significant deviation (Shapiro–Wilk p=0.144). No outliers with absolute standardized residuals greater than 3 were detected, supporting robust inference based on effect sizes and 95% CI.

For the fermentation 9 d D-stage umami scores from samples D-1 to D-6, each sample was evaluated once by 10 panelists (n=60). A linear mixed-effects model was fitted to characterize the

repeated-measures structure. Sample was treated as a fixed effect, and panelist was treated as a random intercept to control baseline scoring differences. A significant overall sample effect was observed (Wald $\chi^2$ (5) =120.32, $p=2.68\times10^{-24}$). The estimated marginal means (95% CI) were 4.00 for D-1 (3.24–4.76), 4.00 for D-2 (3.24–4.76), 3.90 for D-3 (3.14–4.66), 7.30 for D-4 (6.54–8.06), 7.80 for D-5 (7.04–8.56), and 7.10 for D-6 (6.34–7.86). Marked differentiation in umami intensity was observed across samples at this stage. The highest score was obtained for D-5, whereas the lowest score was obtained for D-3. The largest contrast was observed for D-5 versus D-3, with a mean difference of $\Delta=3.90$ points (95% CI 2.83–4.97). After standardization by the model residual SD of 1.22, the effect size was d=3.19, indicating very strong practical relevance in addition to statistical significance. The estimated panelist random-effect variance approached 0 ($\sigma^2$_panelist$\approx2.45\times10^{-10}$), corresponding to ICC<0.001. Model fit was not improved by including the panelist random intercept (likelihood ratio test $\chi^2$ (1) =0.00, p=1.00), indicating negligible systematic panelist bias. Based on common definitions of marginal and conditional $R^2$, the marginal $R^2$ was approximately 0.671 and the conditional $R^2$ was approximately 0.671, indicating that overall variance was largely explained by sample differences. Residual diagnostics indicated good homoscedasticity (Levene test p=0.853). No outliers with absolute standardized residuals greater than 3 were detected. A mild deviation from residual normality was detected under strict testing (Shapiro–Wilk p=0.029). Therefore, effect sizes and their 95% CI were considered the primary reporting basis for inference and presentation.
