# Supplementary material for: Molecular Informatics, Chemometrics, and Sensory Omics for Constructing an Umami Peptide Cluster Library Across the Entire Lager Beer Brewing Process
Source: Foods. 2026 Feb 10;15(4):641. doi: 10.3390/foods15040641 (PMC12939766; doi:10.3390/foods15040641)
Supplement: Supplementary file 1 [file foods-15-00641-s001.zip › Supplementary File S4 Database of Umami Taste Peptide Clusters in the Complete Lager....html]

Lager beer brewing full-process umami peptide cluster database 

# Lager beer brewing full-process umami peptide cluster database

Designed by Wu Yashuai · Supervisor Zhao Dongrui

## Login

  
Login

## Search Umami Peptide

Search
