# Supplementary material for: Molecular Informatics, Chemometrics, and Sensory Omics for Constructing an Umami Peptide Cluster Library Across the Entire Lager Beer Brewing Process
Source: Foods. 2026 Feb 10;15(4):641. doi: 10.3390/foods15040641 (PMC12939766; doi:10.3390/foods15040641)
Supplement: Supplementary file 1 [file foods-15-00641-s001.zip › Supplementary File S5 Database of Umami Taste Peptide Clusters in the Complete Lager Beer....pdf]

Lager beer brewing full-process umami peptide cluster database

Designed by Wu Yashuai · Supervisor Zhao Dongrui

Search Umami Peptide

ATLIDPKRGHVG

Search

Sample ID	-10LgP	Mass	Length	m/z	RT	Area	PTM	ALC (%)	UMPred-FRL-Probability	ProUmami
3-1	18.22	1262.7095	12	421.91097	14.7403	1450			0.974	0.97645

Further analysis

Descriptor	Value
SMILES	CC[C@H](C)[C@H](NC(=O)[C@H](CC(C)C)NC(=O)[C@@H](NC(=O)[C@H](C)N)[C@@H](C)O)C(=O)N[C@@H](CC(=O)O)C(=O)N1CCC[C@H]1C(=O)N[C@@H](CCCCN)C(=O)N[C@@H](CCCNC(=N)N)C(=O)NCC(=O)N[C@@H](Cc1c[nH]cn1)C(=O)N[C@H](C(=O)NCC(=O)O)C(C)C
MinEStateIndex	-1.79873
SMR_VSA1	77.64857
SMR_VSA2	5.40928
SMR_VSA3	73.35254
SMR_VSA4	34.95472
SMR_VSA5	192.54131

Descriptor	Value
SMR_VSA6	32.72378
SMR_VSA7	18.21809
SMR_VSA8	0
SMR_VSA9	0
SMR_VSA10	82.87714
BCUT2D_MWLOW	9.95676
BCUT2D_MWHI	16.37054
VSA_EState1	0
VSA_EState2	182.37087
VSA_EState3	64.4457
VSA_EState4	17.18177
VSA_EState5	-14.26096
VSA_EState6	-13.79867
VSA_EState7	1.38773
VSA_EState8	11.34022
VSA_EState9	0
VSA_EState10	0
EState_VSA1	174.78611
EState_VSA2	89.29912

Descriptor	Value
EState_VSA3	12.11475
EState_VSA4	4.89991
EState_VSA5	26.37164
EState_VSA6	41.54242
EState_VSA7	0
EState_VSA8	68.45263
EState_VSA9	27.71681
EState_VSA10	72.54204
EState_VSA11	0
PEOE_VSA1	100.88915
PEOE_VSA2	67.73827
PEOE_VSA3	4.98398
PEOE_VSA4	0
PEOE_VSA5	0
PEOE_VSA6	47.96324
PEOE_VSA7	89.51252
PEOE_VSA8	25.70718
PEOE_VSA9	37.13263
PEOE_VSA10	60.92132

Descriptor	Value
PEOE_VSA11	5.95955
PEOE_VSA12	64.97898
PEOE_VSA13	0
PEOE_VSA14	11.93861
MolLogP	-5.51823

Further analysis

From the perspective of a transformer-based neural network, the 12-residue peptide "ATLIDPKRGHVG" exhibits a predicted umami probability of 0.976 and likely contributes a savory, brothy depth to lager beer. Its amino acid composition enhances mouthfeel and body, softening the hop-derived bitterness while balancing malt sweetness. Hydrophobic and hydrophilic residues may interact with volatile compounds to release subtle cereal and yeasty aromas, adding complexity and promoting a lingering finish. Overall, this peptide enriches the beer’s sensory profile, delivering roundness and umami harmony without overpowering the refreshing crisp character typical of lagers.
